# Brain-inspired reservoir computing framework for complex time-series prediction

**DOI:** 10.1016/j.isci.2026.116719

**Published:** 2026-07-23

**Authors:** Pei Ma, Xubin Wang, Hanshuo Qiu, Jizhao Liu, Yide Ma, Ludovico Minati

**Affiliations:** 1 School of Information Science and Engineering, Lanzhou University, Lanzhou, Gansu 730000, China; 2 National Key Laboratory on Vacuum Technology and Physics, Lanzhou Institute of Physics, Lanzhou, Gansu 730000, China; 3 School of Electronics and Communication Engineering, Lanzhou University of Arts and Sciences, Lanzhou, Gansu 730000, China; 4 School of Life Science and Technology, University of Electronic Science and Technology of China, Chengdu, Sichuan 610054, China; 5 Department of Physics, University of Trento, 38123 Trento, Italy

**Keywords:** BINN-ESN, CCNN, time series prediction, reservoir computing, brain-inspired computing

## Abstract

Time-series prediction based on historical data is essential in numerous scientific fields, such as weather prediction and financial markets analysis. However, obtaining strong predictive accuracy together with high computational efficiency remains challenging. To address this challenge, we propose a brain-inspired neural network-based echo state network (BINN-ESN). It uses a modified continuous coupled neural network (MCCNN) as the neural model, which is inspired by the mammalian visual cortex. Our results indicate a system-dependent trade-off: While deep learning baselines generally achieve stronger single-step accuracy, BINN-ESN shows stronger long-term predictive stability on most evaluated systems and requires substantially less total computation than the GPU-accelerated long short-term memory (LSTM) baseline in long-term experiments. Our code is available at https://github.com/Jizhao-Liu/code-for-BINN-ESN.

## Highlights

• We improved the CCNN model and designed the corresponding reservoir computing framework
• We observe that BINN-ESN achieves a favorable balance between performance and efficiency
• We show neurons exhibit digital-like mode behavior in functions despite complex dynamics

## Graphical abstract

**Figure:**
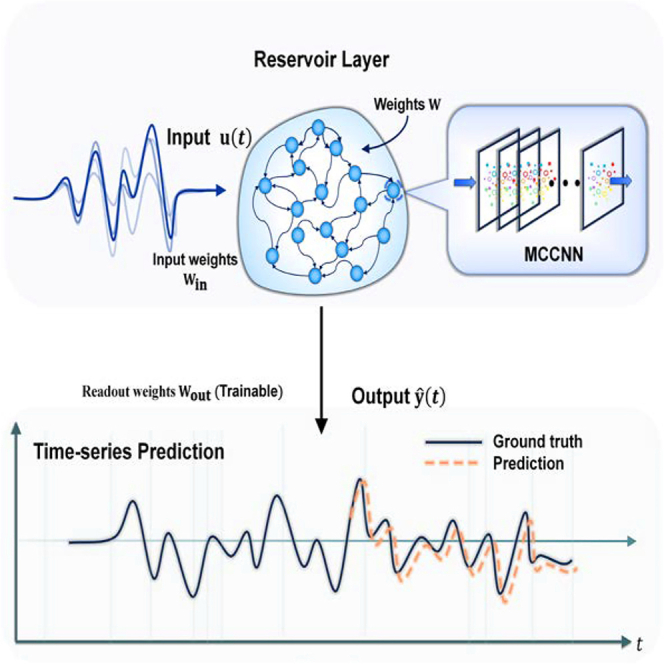

## Teaser

Neural networks; Applied sciences; Machine learning; Computer network

## Introduction

Time series forecasting is an important task in many scientific and technical fields,^1^ including financial market analysis^2^ and weather forecasting.^3^ The intricate temporal dependencies present in systems governed by nonlinear and often chaotic dynamics are poorly captured by traditional linear models, such as the integrated moving average autoregressive model (ARIMA).^1^

Besides, the advent of recurrent neural networks (RNNs), and in particular the long short-term memory (LSTM) architecture, has advanced state-of-the-art approaches.^4^ In fact, by using advanced gating mechanisms, LSTM networks can effectively model long-term dependencies. However, their performance comes at a considerable cost, due to the complexity of their architecture and the large number of trainable parameters, leading to high computational requirements and an increased risk of overfitting. This is particularly observed in scenarios where data are limited.^5^

At the other end of the spectrum, reservoir computing (RC), which is exemplified by echo state networks (ESNs), offers a computationally efficient paradigm.^6^ ESNs utilize a fixed, randomly generated recurrent network—the “reservoir”—and only train a linear readout layer, which drastically reduces the required training time. However, this simplicity presents its own limitations. Their reliance on simple static activation functions (e.g., tanh) restricts the network’s memory capacity to the global topology of the reservoir (i.e., the spectral radius). This creates a fundamental bottleneck: Standard ESNs lack intrinsic multi-timescale memory processing at the node level, making them highly vulnerable to exponential error accumulation when forecasting chaotic dynamics over long horizons.^7^ Furthermore, advanced ESN variants (e.g., deep ESNs) attempt to solve this by stacking architectural layers, which significantly inflates hyperparameter complexity without altering the fundamental limitations of the static node. This presents a critical tradeoff between the high performance but costly complexity of LSTM networks and the high efficiency but limited representational power of ESNs. We propose that a promising path for resolving this tradeoff lies in drawing inspiration from computational neuroscience. Instead of expanding the macroscopic network topology, we argue that the fundamental unit of computation should be upgraded: substituting the static activation function with a bio-inspired dynamic kernel that transforms each node into an independent nonlinear dynamical sub-system, enriching the reservoir’s representational phase space without expanding the gradient-trained parameter scale.^8^^,^^9^

The central idea of this work stems from the previous observation. By replacing the static activation function of an ESN with a “dynamic kernel” that emulates the computational richness of a biological neuron, it is possible to create a model that achieves a compelling balance between performance and efficiency. This work, which thus introduces a brain-inspired neuron network-based echo state network (BINN-ESN), has led to the following contributions.

1. We design and implement a modified continuous coupled neural network (MCCNN), a recurrent neuron with complex internal dynamics inspired by the mammalian visual cortex, and a BINN-ESN model that is integrated into the efficient RC paradigm.
2. Through a statistically sound experimental protocol, we observe that BINN-ESN achieves a favorable balance between performance and efficiency: Deep learning baselines are typically stronger in single-step accuracy, whereas BINN-ESN attains the highest mean valid prediction time (VPT) on most evaluated systems and remains more computationally efficient than GPU-accelerated deep learning networks (LSTM, temporal convolutional network (TCN), and Transformer) in long-term prediction experiments (see Section computational efficiency analysis for detailed efficiency analysis).
3. We conduct a systematic investigation showing that the model’s performance relies on its ability to self-organize into a robust, “digital-like” computational mode, directly stemming from its bio-inspired design.

## Results

### Related works

This work lies at the intersection of three major areas, namely deep learning applied to time series forecasting, RC, and brain-inspired neuronal modeling. In this section, we review the most advanced approaches in each of these fields to situate more precisely our contribution.

#### Deep learning approaches for time series forecasting

With the advent of deep learning, time series prediction methodologies have increasingly shifted toward architectures based on neural networks capable of capturing complex and nonlinear dependencies. The classic RNNs represented a major advancement in processing temporal information thanks to recurrent connections. However, their practical use remains hindered by the well-known problems of vanishing and exploding gradients, which limit their ability to model long-term dependencies.^10^

To address these issues, the LSTMs were introduced. The latter incorporate a memory cell controlled by input, forgetting, and output gates,^4^ allowing them to selectively retain and delete information over long periods, making it highly effective for a wide range of forecasting tasks. Mathematically, the core computation within an LSTM cell at time step *t* is governed by the following equations, which control the flow of information via the forget gate (*f*_*t*_), input gate (*i*_*t*_), and output gate (*o*_*t*_):

(Equation 1) $$\left\{\begin{array}{cc} f_{t} & =\sigma \left(W_{f}\cdot \left[h_{t-1},x_{t}\right]+b_{f}\right)\\ i_{t} & =\sigma \left(W_{i}\cdot \left[h_{t-1},x_{t}\right]+b_{i}\right)\\ {\tilde{C}}_{t} & =\tanh\left(W_{C}\cdot \left[h_{t-1},x_{t}\right]+b_{C}\right)\\ C_{t} & =f_{t}⊙C_{t-1}+i_{t}⊙{\tilde{C}}_{t}\\ o_{t} & =\sigma \left(W_{o}\cdot \left[h_{t-1},x_{t}\right]+b_{o}\right)\\ h_{t} & =o_{t}⊙\tanh\left(C_{t}\right) \end{array}\right)$$

where *σ* represents the sigmoid activation function, ⊙ denotes element-wise multiplication, **x**_*t*_ is the input, **h**_*t*−1_ is the hidden state, and *C*_*t*_ is the cell state. Beyond their streamlined design, variants such as recurrent gate units (GRUs) have proven to be powerful.^11^ To overcome the sequential computation bottleneck of RNNs, TCNs have been introduced, utilizing dilated causal convolutions to achieve exponentially large receptive fields. The core dilated convolution operation F on sequence **x** at time step *t* is defined as:

(Equation 2) $$F\left(t\right)=\left({x*}_{d}f\right)\left(t\right)=\overset{k-1}{\underset{i=0}{\sum }}f\left(i\right)\cdot x_{t-d\cdot i}$$

where *d* is the dilation factor, *k* is the filter size, and *f* represents the convolution filter.

More recently, Transformer architectures have stood out for their impressive performance, particularly on large datasets where their ability to process data in parallel is fully leveraged.^12^ The fundamental mechanism enabling this is the scaled dot-product attention, which dynamically weighs the relevance of historical time steps:

(Equation 3) $$\text{Attention}\left(Q,K,V\right)=\text{softmax}\left(\frac{QK^{T}}{\sqrt{d_{k}}}\right)V$$

where *Q*, *K*, and *V* denote the query, key, and value matrices projected from the time-series embedding, and *d*_*k*_ is the dimension of the keys. Building upon this mathematical foundation, specialized architectures such as Informer,^13^ Autoformer,^14^ and PatchTST^15^ have successfully adapted the Transformer mechanism specifically for long-term time-series forecasting by introducing sparse attention, series decomposition, and channel-independent patching, respectively.

Although these deep learning approaches have achieved success, they share the common characteristic of substantial computational requirements and precise tuning. Furthermore, due to the high number of trainable parameters, the risk of overfitting increases in scenarios with limited data or low noise, such as the benchmark dynamic systems used in this study. This trade-off between high performance and high cost has fostered the exploration of more efficient computational paradigms.^5^^,^^16^

#### Reservoir computing

RC emerged as a paradigm for addressing the challenges of training RNNs by simplifying the learning process.^17^ An ESN, the most prominent type of RC model, uses a large, sparsely connected RNN—the “reservoir”—whose connection weights are randomly generated and remain fixed.^18^ The only trainable component is a linear readout layer that learns to map the high-dimensional reservoir states to the desired output. Formally, the state update and output equations of a standard leaky-ESN at time *t* are defined as:

(Equation 4) $$\left\{\begin{array}{cc} \tilde{x}\left(t\right) & =\tanh\left(W_{\text{in}}\left[1;u\left(t\right)\right]+Wx\left(t-1\right)\right)\\ x\left(t\right) & =\left(1-a\right)x\left(t-1\right)+a\tilde{x}\left(t\right)\\ y\left(t\right) & =W_{\text{out}}\left[1;u\left(t\right);x\left(t\right)\right] \end{array}\right)$$

where **u**(*t*) is the input, **x**(*t*) is the reservoir state, $a\in \left(\left(0,1\right)\right]$ is the leakage rate, **W**_in_ and **W** are the fixed input and recurrent weight matrices, and **W**_out_ is the trainable readout matrix. This approach results in exceptionally fast learning processes by bypassing the difficult gradient-based training procedures required for recurrent weights.

The performance of an ESN relies on precise tuning of its hyperparameters so that the reservoir evolves into a stable state that is rich enough for computation. This behavior is known as the echo state property.^6^ Key parameters include the reservoir size, the spectral radius of the recurrent weight matrix, and its sparsity. The sensitivity of the network to these parameters requires extensive trial-and-error or computationally expensive optimization searches.^19^^,^^20^

Numerous extensions have been proposed to enhance the capabilities of ESNs. These methods include introducing a leakage rate to control the speed of the dynamics of neurons (leaky ESN),^6^ using multiple parallel reservoirs to process information at different time scales,^21^ and stacking reservoirs to form deep hierarchical structures (deep ESN).^22^ Other innovative approaches strengthen memory mechanisms through fractional-order calculus techniques^23^ or by introducing specialized topologies such as double-loop ESNs,^24^ long-short-term ESNs,^25^ and brain-inspired small-world reservoir topologies.^26^^,^^27^ For instance, incorporating a small-world topology has been proven to significantly enhance the reservoir’s dynamic properties and signal propagation by mimicking the highly clustered yet short-path connectivity of human cortical networks.^26^ Although these advanced architectures have pushed the performance boundaries of RC, they usually increase architectural complexity and still fundamentally rely on neurons with simple static activation functions (e.g., tanh). This fundamental simplification at the neuron level may ultimately limit the ability of the reservoir to fully capture the richness of complex chaotic dynamics.

#### Brain-inspired neuronal modeling in reservoir computing scenarios

This work falls within a rapidly growing field of research that draws inspiration from the brain’s computational principles to make artificial neural networks more closely resemble real biological functioning by giving each neuron a richer and more adaptive internal behavior.

A prominent example of this approach is the pulse-coupled neural network (PCNN) proposed by Eckhorn and his collaborators,^28^^,^^29^ which is derived from bio-inspired models of the mammalian visual cortex. The latter and its variants have demonstrated significant potential in image processing tasks such as segmentation, fusion, and feature extraction^30^ thanks to their ability to simulate complex neuronal phenomena such as synchronous pulse bursts and dynamic capture behavior. Johnson et al.^31^ later proposed a formal mathematical model of the PCNN, describing the internal state of neurons through a set of coupled differential equations, including fed input, linked input, and dynamic threshold.

Over the years, the PCNN model has inspired a rich family of derivative and simplified models. These include the intersecting cortical model (ICM),^32^ the spiking cortical model (SCM),^33^ and various simplified PCNNs (SPCNNs),^34^ each of which is aimed at providing improved performance, reducing the parameter complexity level, or tailoring the developed model for specific applications. Recently, the continuous coupled neural network (CCNN) capable of reproducing complex nonlinear behaviors observed in biological neurons was proposed by Liu et al.^9^ as an extension. By modeling neural excitation as a continuous and stochastic process, the CCNN manages to generate periodic outputs in response to direct current (DC) stimuli and chaotic dynamics under alternating current (AC) stimulation.

While the majority of the aforementioned brain-inspired models have been developed and applied for image processing and computer vision, we argue that their true value lies in their underlying *dynamical principles*. The present work abstracts these powerful bioinspired dynamics into a generalized, efficient computational unit to create a new class of ESNs. Unlike methods that primarily alter the network’s overall structure, this work’s approach focuses on improving the behavior of each neuron to transform neuronal mechanisms into simple and efficient computational units.

### Performance comparison with baseline models

The BINN-ESN, standard ESN, LSTM, TCN, and Transformer are evaluated and compared in terms of short-term predictive accuracy and long-term stability under a unified protocol across five systems (Lorenz, Chen, Lü, Mackey-Glass, and ETTh1). A dedicated efficiency analysis is provided in Section computational efficiency analysis.

#### Short-term prediction accuracy

The fundamental capability of any forecasting model is its accuracy in one-step-ahead prediction tasks. All five optimized models are evaluated on this task, with the statistical results obtained over 10 trials and summarized in Table 1.

**Table 1:** Short-term prediction performance comparison

System	Model	Mean squared error (MSE)	Mean absolute error (MAE)
Lorenz	BINN-ESN	1.0518e-03 ± 1.2290e-03	3.1719e-03 ± 2.1032e-03
	ESN	1.3681e-03 ± 2.3130e-03	2.7826e-03 ± 2.5706e-03
	LSTM	2.4622e-04 ± 1.3739e-04	8.7236e-03 ± 7.6077e-04
	TCN	3.5287e-04 ± 1.9665e-04	1.2256e-02 ± 2.4146e-03
	transformer	1.0465e-03 ± 5.1836e-04	2.3027e-02 ± 6.6505e-03
Chen	BINN-ESN	1.9863e-03 ± 2.7188e-03	5.8230e-03 ± 4.1289e-03
	ESN	2.9124e-03 ± 5.7215e-03	3.9466e-03 ± 3.2401e-03
	LSTM	4.5885e-04 ± 4.0418e-04	9.5634e-03 ± 2.5549e-03
	TCN	4.2387e-04 ± 2.8926e-04	1.2103e-02 ± 3.5649e-03
	transformer	1.0621e-03 ± 9.8926e-04	1.9865e-02 ± 7.5797e-03
Lü	BINN-ESN	1.4422e-03 ± 1.7698e-03	4.9887e-03 ± 4.6490e-03
	ESN	1.8159e-03 ± 2.2412e-03	4.1567e-03 ± 2.4370e-03
	LSTM	1.0932e-03 ± 2.0616e-03	1.0369e-02 ± 5.3535e-03
	TCN	8.6568e-04 ± 1.4316e-03	1.2530e-02 ± 4.6233e-03
	transformer	1.3582e-03 ± 1.4761e-03	2.1827e-02 ± 6.3921e-03
Mackey-Glass	BINN-ESN	1.1118e-04 ± 1.7329e-04	1.3642e-03 ± 1.1931e-03
	ESN	5.8269e-05 ± 6.4455e-05	1.1803e-03 ± 6.6535e-04
	LSTM	7.6143e-07 ± 1.4701e-07	6.5746e-04 ± 6.6337e-05
	TCN	2.5970e-06 ± 2.8990e-06	1.1709e-03 ± 6.0768e-04
	transformer	1.6748e-06 ± 1.0526e-06	9.8013e-04 ± 3.4961e-04
ETTh1	BINN-ESN	7.9837e+03 ± 1.7515e+04	8.1393e+00 ± 1.0814e+01
	ESN	1.4572e+04 ± 2.3525e+04	4.4613e+00 ± 3.4851e+00
	LSTM	4.3195e-01 ± 9.4582e-02	4.7047e-01 ± 6.7696e-02
	TCN	4.0543e-01 ± 1.2744e-02	4.5321e-01 ± 1.0674e-02
	transformer	4.2561e-01 ± 3.6575e-02	4.6695e-01 ± 2.6192e-02

The results are mean ± std. dev. values calculated over 10 trials. The best performance achieved in terms of each metric on each system is in bold.

For clarity, the hyperparameter search objective is the validation single-step MSE defined in Equation 17 (Section automated hyperparameter optimization), and the long-term free-running evaluation reuses the model parameters optimized in this short-term stage without additional retraining.

**Figure 1:**
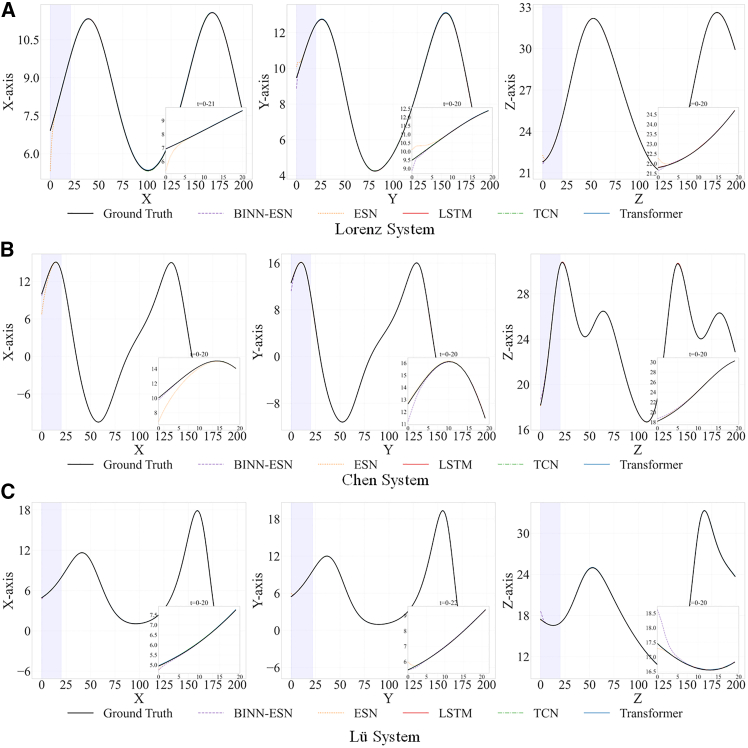
Qualitative comparison among single-step prediction trajectories over a 200-step local zoom window selected from hard-to-fit segments Each panel corresponds to a 3D chaotic system: (A) Lorenz, (B) Chen, and (C) Lü. The black solid line denotes the ground truth, and the other curves denote model predictions.

**Figure 2:**
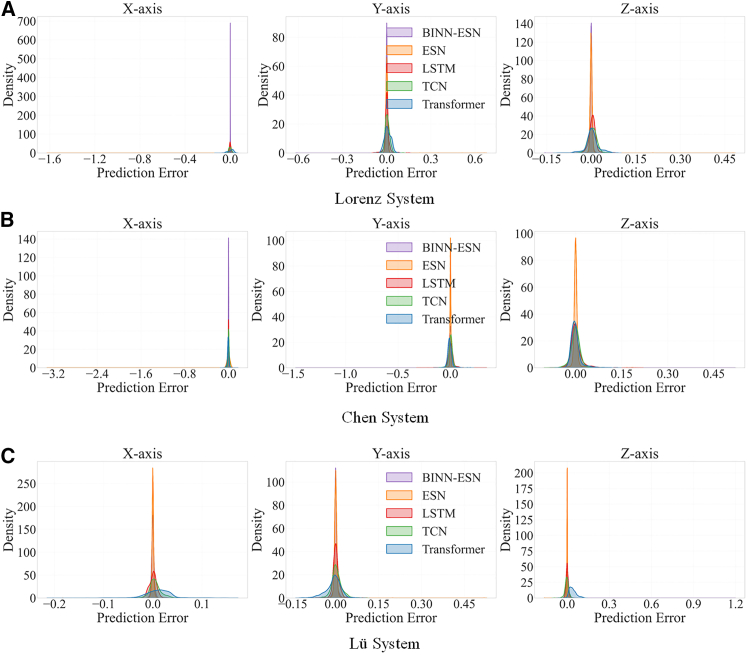
Probability density distributions of single-step prediction errors (Prediction - True) across the three 3D chaotic systems Each panel corresponds to (A) Lorenz, (B) Chen, and (C) Lü, with x/y/z-axis error densities shown in each panel. Mackey-Glass and ETTh1 are not included here because they are univariate and therefore do not admit comparable multi-axis density visualization.

Table 1 shows a system- and metric-dependent pattern. For the three 3D chaotic systems, the lowest MSE is achieved by LSTM on Lorenz (2.4622e-04) and by TCN on Chen (4.2387e-04) and Lü (8.6568e-04), while ESN gives the lowest MAE on all three systems (2.7826e-03, 3.9466e-03, and 4.1567e-03, respectively). BINN-ESN remains numerically competitive with ESN in these three systems (e.g., Lorenz MSE 1.0518e-03 vs. 1.3681e-03; Chen MSE 1.9863e-03 vs. 2.9124e-03; Lü MSE 1.4422e-03 vs. 1.8159e-03), while deep-learning baselines are stronger on the expanded benchmarks: On Mackey-Glass, LSTM attains the best MSE/MAE (7.6143e-07/6.5746e-04), and on ETTh1, TCN attains the best MSE/MAE (4.0543e-01/4.5321e-01). Because Mackey-Glass and ETTh1 are univariate targets, multivariate trajectory overlays and axis-wise error-distribution plots are only reported for the three 3D chaotic systems in Figures 1 and 2. The error-density plots suggest that BINN-ESN exhibits a relatively higher error peak in Lorenz, whereas in the other displayed systems its distributions appear comparatively concentrated. Figure 1 presents local zooms on hard-to-fit segments; in the shown examples, deep-learning models often exhibit tighter local alignment, while BINN-ESN and ESN can show larger initial mismatch and then move closer to the ground truth later in the window.

For system-specific visual references, trajectory overlays are shown in Figures 1A–1C, while axis-wise error-density panels are shown in Figures 2A–2C.

These observations are consistent with model capacity and objective-function alignment. In our short-term setting, the primary task is single-step prediction, and gradient-trained deep architectures are directly optimized for the one-step MSE objective; therefore, their advantage in this setting is expected despite higher training cost. At the same time, relative to the standard ESN, BINN-ESN achieves lower one-step MSE on most evaluated systems under the unified protocol, indicating that the introduced bio-inspired neuron dynamics improve short-term predictive precision without changing the reservoir-computing training paradigm.

A detailed efficiency analysis is provided in Section computational efficiency analysis.

#### Long-term stability analysis

Although single-step accuracy is essential, it is the long-term stability in a free-running recursive prediction mode that mainly characterizes a reliable model for chaotic systems. Unless otherwise stated, each long-term free-running evaluation is conducted over a fixed horizon of 2000 recursive prediction steps. Here, we evaluate this using two main metrics: the multistep normalized root-mean-square error (NRMSE) and the VPT. These are further complemented by the analysis of the total computational cost, which plays a significant role in the overall assessment of the model.

The results presented in Table 2 show clear system dependence across the five-system benchmark. BINN-ESN attains the highest mean VPT on most evaluated systems, indicating a robust long-term stability advantage in the majority of settings. At the same time, the method is not universally optimal on every dataset. As summarized in Table 2, pairwise differences should be interpreted together with the observed cross-seed variances, which are reported as standard deviations across the 10 independent trials.^35^

**Table 2:** Long-term summary in terms of valid prediction time in steps (VPT Steps) and Lyapunov times (VPT LT)

System	Model	VPT steps (mean ± std)	VPT LT (mean ± std)
Chen	BINN-ESN	595.60 ± 743.19	6.036 ± 7.532
	ESN	510.70 ± 537.14	5.176 ± 5.444
	LSTM	467.50 ± 548.66	4.738 ± 5.561
	TCN	348.50 ± 423.42	3.532 ± 4.291
	transformer	120.40 ± 98.47	1.220 ± 0.998
ETTh1	BINN-ESN	2.20 ± 1.87	N/A
	ESN	5.00 ± 4.55	N/A
	LSTM	12.00 ± 4.35	N/A
	TCN	9.90 ± 4.58	N/A
	transformer	6.60 ± 3.27	N/A
Lorenz	BINN-ESN	868.10 ± 483.67	3.931 ± 2.190
	ESN	588.10 ± 416.94	2.663 ± 1.888
	LSTM	411.90 ± 327.54	1.865 ± 1.483
	TCN	426.40 ± 348.78	1.931 ± 1.579
	transformer	139.30 ± 111.25	0.631 ± 0.504
Lü	BINN-ESN	435.80 ± 160.09	2.902 ± 1.066
	ESN	545.20 ± 324.48	3.630 ± 2.160
	LSTM	441.00 ± 257.32	2.936 ± 1.713
	TCN	209.00 ± 147.80	1.392 ± 0.984
	transformer	83.90 ± 50.58	0.559 ± 0.337
Mackey-Glass	BINN-ESN	434.00 ± 377.57	2.604 ± 2.265
	ESN	354.90 ± 311.48	2.129 ± 1.869
	LSTM	339.10 ± 157.22	2.035 ± 0.943
	TCN	254.30 ± 140.91	1.526 ± 0.845
	transformer	245.00 ± 119.22	1.470 ± 0.715

The results are reported as mean ± std. dev. values over 10 trials.

The trade-off between this high performance and the incurred computational cost is analyzed in Section computational efficiency analysis.

**Figure 3:**
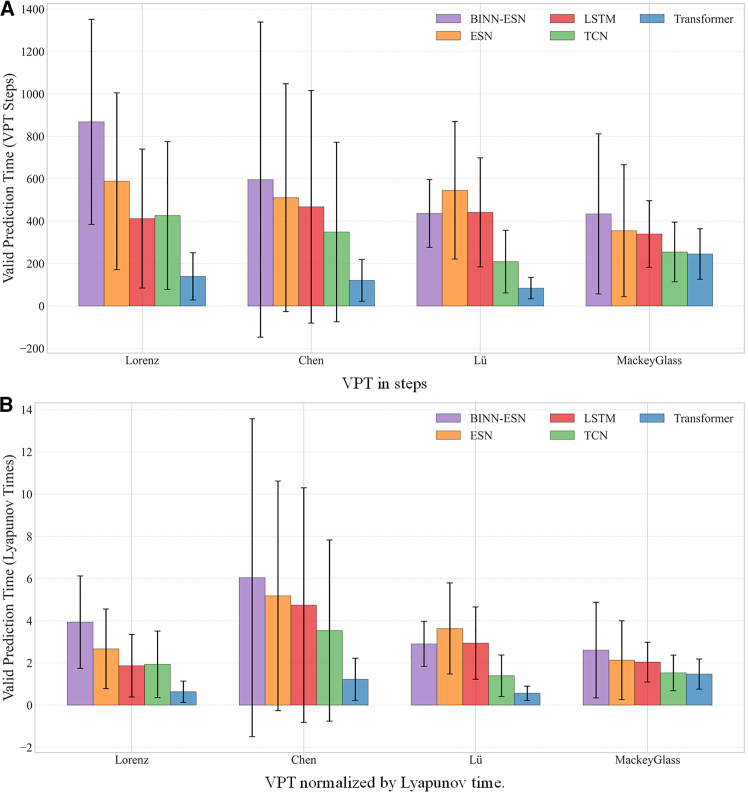
Long -term predictive stability comparison Long-term predictive stability comparison across models under two complementary scales: (A) mean valid prediction time in steps (VPT Steps) with standard deviation error bars, and (B) mean valid prediction time normalized by Lyapunov time (VPT LT) with standard deviation error bars. Note that ETTh1 is omitted from this figure because its VPT values are orders of magnitude smaller than those of the chaotic systems, making it invisible on the same scale.

**Figure 4:**
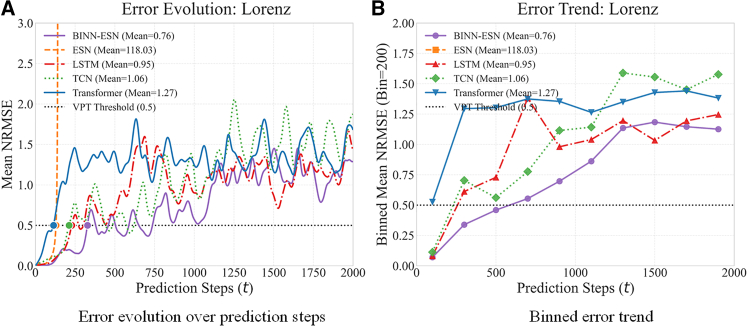
Long-term NRMSE analysis for the Lorenz system (A) Error evolution over prediction steps. (B) Binned error trend (bin size = 200). The horizontal dotted line indicates the VPT threshold (NRMSE = 0.5).

**Figure 5:**
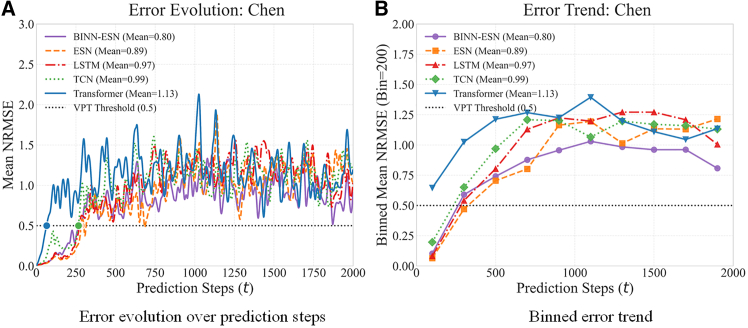
Long-term NRMSE analysis for the Chen system (A) Error evolution over prediction steps. (B) Binned error trend (bin size = 200). The horizontal dotted line indicates the VPT threshold (NRMSE = 0.5).

**Figure 6:**
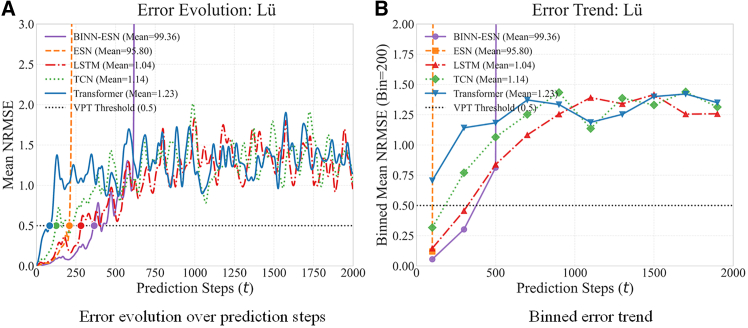
Long-term NRMSE analysis for the Lü system (A) Error evolution over prediction steps. (B) Binned error trend (bin size = 200). The horizontal dotted line indicates the VPT threshold (NRMSE = 0.5).

**Figure 7:**
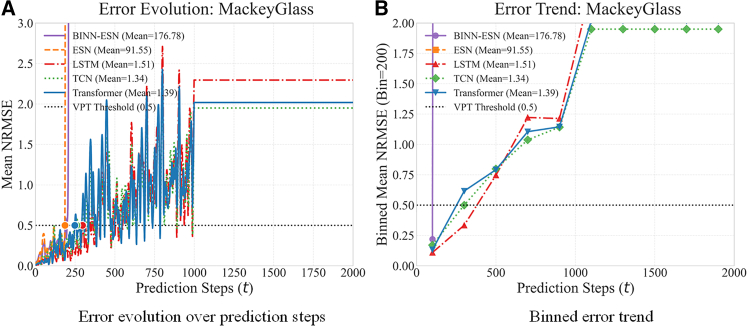
Long-term NRMSE analysis for the Mackey-Glass system Left: error evolution over prediction steps. Right: binned error trend (bin size = 200). The horizontal dotted line indicates the VPT threshold (NRMSE = 0.5).

**Figure 8:**
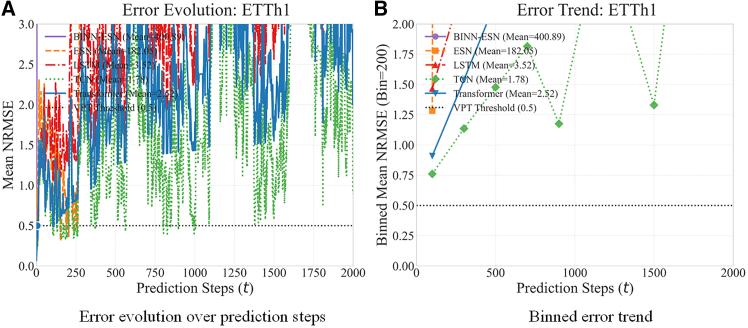
Long-term NRMSE analysis for the ETTh1 dataset Left: error evolution over prediction steps. Right: binned error trend (bin size = 200). The horizontal dotted line indicates the VPT threshold (NRMSE = 0.5).

The detailed dynamics of error accumulation are shown in Figures 4, 5, 6, 7, and 8, together with Figure 3, and are quantitatively summarized in Table 2. A clear system-dependent pattern emerges. In VPT Steps, BINN-ESN attains the highest mean values on Lorenz (868.10 ± 483.67), Chen (595.60 ± 743.19), and Mackey-Glass (434.00 ± 377.57), whereas ESN is highest on Lü (545.20 ± 324.48) and LSTM is highest on ETTh1 (12.00 ± 4.35). The same ranking trend is largely preserved after Lyapunov-time normalization: BINN-ESN is highest on Lorenz (3.931 ± 2.190), Chen (6.036 ± 7.532), and Mackey-Glass (2.604 ± 2.265), while ESN is highest on Lü (3.630 ± 2.160), confirming that no single model is uniformly optimal across all systems.

Specifically, Figure 3A reports VPT in steps, and Figure 3B reports VPT normalized by Lyapunov time.

Figures 4, 5, 6, and 7 further provide trajectory-level evidence. In the Chen and Lorenz panels, the BINN-ESN curves are predominantly located at the lower envelope of the five-model set in both the raw evolution and binned-trend views, indicating better average long-horizon error control. In contrast, for Lü and Mackey-Glass, both ESN-family models (ESN and BINN-ESN) exhibit sharp NRMSE escalation within roughly the first 500 prediction steps; in the raw-evolution subpanels, this escalation is accompanied by values exceeding the displayed y-axis range in part of the horizon. Nevertheless, the onset of this rapid growth is generally delayed for BINN-ESN relative to standard ESN, which is consistent with the higher mean VPT of BINN-ESN on Mackey-Glass in Table 2.

These observations suggest that the classical ESN architecture may not be uniformly suitable across all dynamical regimes, and that BINN-ESN mitigates but does not fully eliminate this limitation on the most difficult systems. ETTh1 is the most challenging benchmark in this analysis: All models show early threshold crossing, strong volatility, and frequent numerical excursions beyond the plotted y-axis range, indicating severe long-horizon instability under the current free-running setup. Finally, the wide error bars in Figure 3 (notably for Chen) indicate substantial cross-seed variability; therefore, robustness should be interpreted jointly from central tendency and dispersion rather than from mean values alone. In particular, because each seed is executed as an independent pipeline (seed-specific trajectory, seed-specific Bayesian search, and seed-specific model initialization/training), the reported standard deviations naturally capture this compounded uncertainty rather than initialization noise alone.

**Figure 9:**
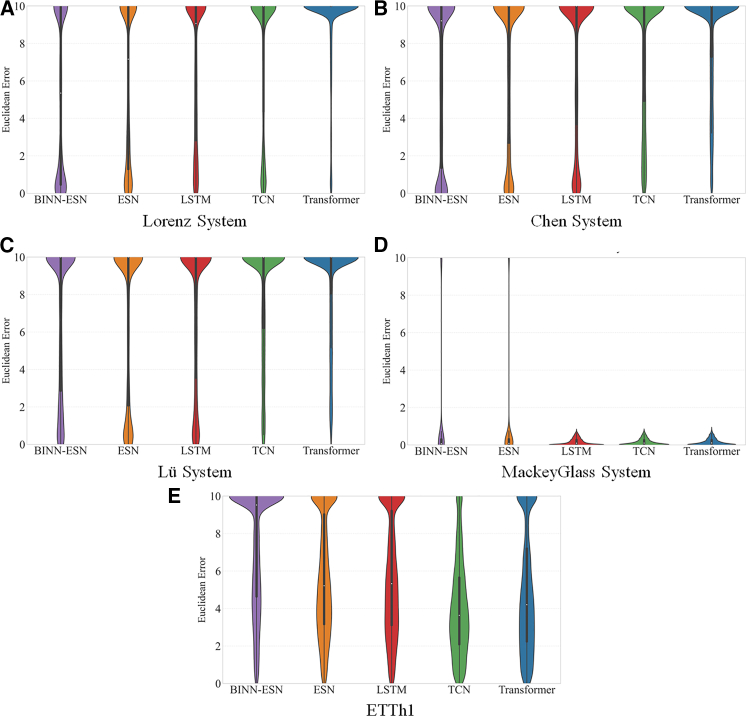
Long-term error distribution (violin plot) across five systems over 2000 prediction steps The Euclidean error is clipped at 10.0 for visualization. Each violin shows the full error density; the embedded box indicates the interquartile range. Lower and more concentrated distributions indicate more consistent long-term prediction behavior.

The error distribution across the full 2000-step horizon is further examined via violin plots (Figure 9). On the Lorenz and Chen systems, BINN-ESN achieves the lowest median error among all models, while all models exhibit substantial mass near the clipping boundary (10.0), reflecting the inherent difficulty of long-horizon chaotic prediction. On the Mackey-Glass system, all models show substantially lower error magnitudes compared to the chaotic attractors, and the median errors are closely grouped; notably, ESN exhibits a kurtosis of 7.31, indicating a heavy-tailed, bimodal distribution in which the model frequently operates near the error floor but occasionally produces large deviations, whereas BINN-ESN shows a kurtosis of 1.66, indicating a more concentrated and consistent error profile. This contrast is not captured by median or mean statistics alone and illustrates why BINN-ESN achieves a higher VPT on this system despite ESN appearing competitive in raw error magnitude. On the Lü system, all models cluster near the upper clipping boundary, with Transformer showing the highest kurtosis (1.27). On ETTh1, all models show elevated error distributions, with TCN attaining the lowest median error (3.64), and BINN-ESN the highest (9.52), consistent with its known difficulty on real-world multivariate data.

#### ACF-based dynamical consistency analysis

To further investigate the dynamical mechanisms underlying the observed long-term stability differences, we perform an ACF-shape consistency analysis. For each system, the autocorrelation function of a model-generated trajectory is compared against that of the ground-truth trajectory over a lag range of 0–200 steps, directly revealing whether a model preserves the system’s intrinsic timescale rather than relying on threshold-based metrics alone. The first 200 lags are the most informative region for assessing short-to-medium-range temporal dependencies in all five systems.

It should be noted that the ACF analysis operates on the first variable (x-component) of each system only. This design choice reflects a deliberate division of analytical labor: The NRMSE analysis in the preceding section already captures cross-dimensional prediction error through the Euclidean norm, whereas the ACF here serves a distinct purpose—to assess whether the model preserves the system’s intrinsic single-variable temporal structure. For the chaotic attractor systems (Lorenz, Chen, Lü, Mackey-Glass), all three components share the same dynamical origin and exhibit strongly coupled autocorrelation structures, making the x-component a reliable proxy for system-level temporal behavior.

**Figure 10:**
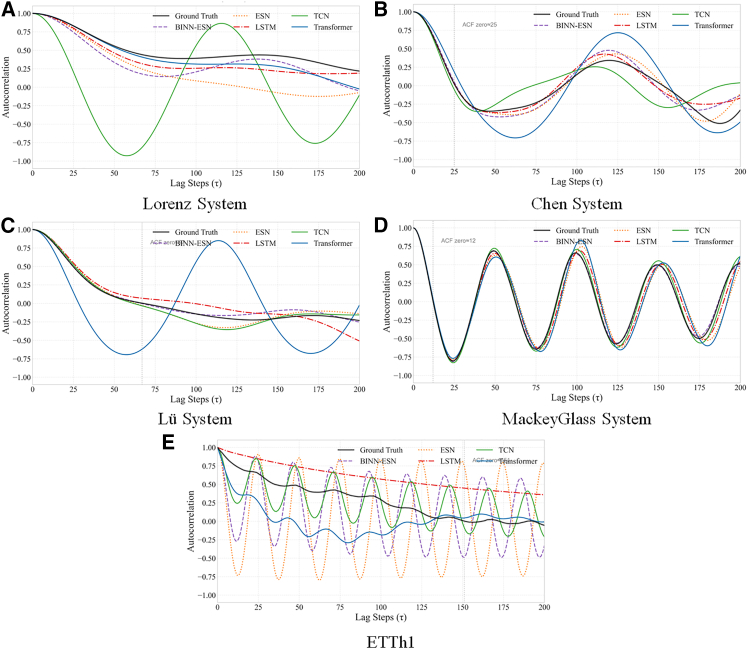
ACF-shape consistency analysis across five systems Each panel compares the autocorrelation decay of model predictions with the corresponding ground-truth trajectory. Closer overlap indicates better preservation of intrinsic temporal dependency structure.

As shown in Figure 10, the five systems exhibit markedly different ACF profiles, reflecting their distinct dynamical characteristics. Lorenz and Lü display oscillatory decay (zero-crossing ≈67 for Lü); Chen decays faster (≈25) with negligible oscillation; Mackey-Glass retains correlations over the remarkably long horizon (zero-crossing ≈12), a defining trait of its delay-feedback dynamics; ETTh1 attenuates most rapidly, consistent with its non-stationary real-world nature.

The model comparison yields system-dependent patterns of varying subtlety. On the Chen system, BINN-ESN, ESN and LSTM all align closely with the ground-truth ACF for the first ∼30 lags; beyond this range, the ACF zero-crossing at lag ≈25 marks the onset of decorrelation, after which TCN and Transformer progressively diverge while BINN-ESN continues to track the ground-truth envelope with minimal deviation. On the Lorenz system, BINN-ESN, ESN, and Transformer all capture the ACF profile well at short lags; ESN exhibits gradually increasing deviation, while TCN deviates substantially throughout and fails to capture the oscillatory decay characteristic. On the Lü system, all models except Transformer achieve close alignment; BINN-ESN shows the best performance, nearly perfectly reproducing the ACF envelope. On the Mackey-Glass system, all models perform well at short lags (before the ACF zero at lag ≈12); BINN-ESN again exhibits the smallest deviation, while all other models show progressively increasing offset with lag, with Transformer deviating most severely. The ETTh1 case stands apart: All models fail to capture the system’s temporal structure. Deep learning baselines (LSTM, TCN, Transformer) produce near-flat ACF curves, a signature of failure to learn any meaningful dynamics rather than a genuine match. This underscores that structural similarity to the ground-truth ACF is not accidental but reflects genuine dynamical reconstruction.

Across all five systems, BINN-ESN demonstrates the strongest and most consistent ability to preserve the intrinsic temporal structure of the target dynamics. Its superiority is most pronounced on Mackey-Glass, where the system’s exceptionally slow decay (zero-crossing ≈12) makes faithful ACF reconstruction particularly challenging. These findings confirm that the superior VPT performance of BINN-ESN is not accidental but rooted in a genuine preservation of the system’s dynamical timescale across a broad range of system characteristics.

#### High-fidelity reconstruction of dynamic structures

In addition to quantitative error metrics, it is important to assess whether a model has truly learned the underlying *dynamic structure* of the target system. This was obtained by analyzing the fidelity with which each model reconstructs the system’s attractor in the phase space, in addition to a more detailed structural comparison conducted using recurrence plots (RPs).

##### Attractor reconstruction in the phase space

**Figure 11:**
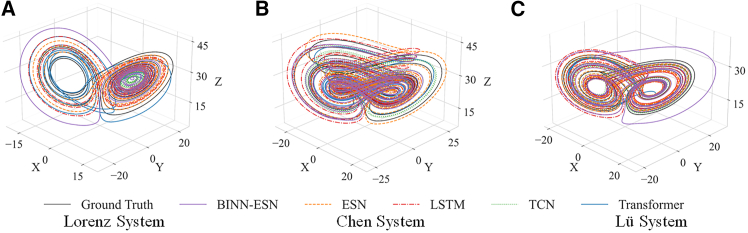
Compact 3D attractor reconstruction from 2000-step free-running predictions Ground truth (black), BINN-ESN (purple), ESN (orange), LSTM (red), TCN (green), Transformer (blue).

**Figure 12:**
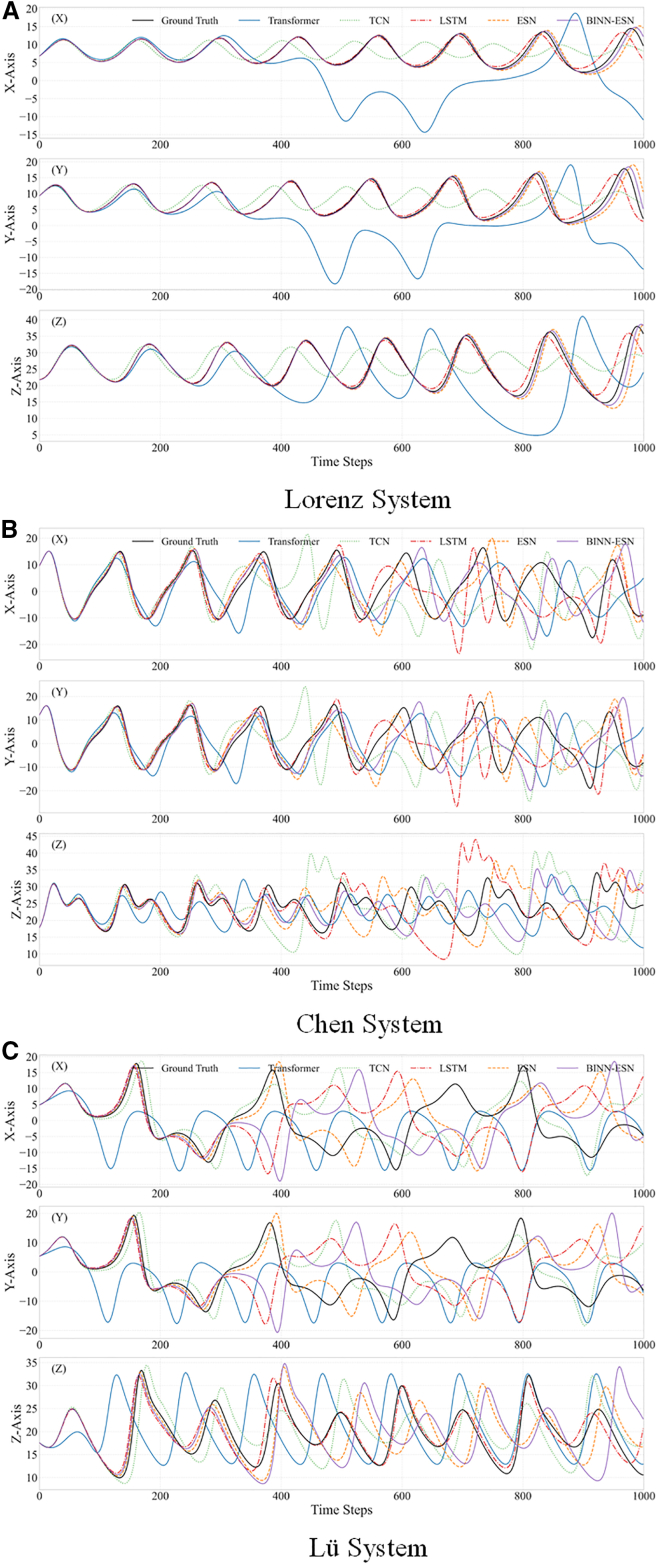
Dimension-wise phase-space trajectories (X, Y, Z) for the three chaotic systems Ground truth is shown in black.

**Figure 13:**
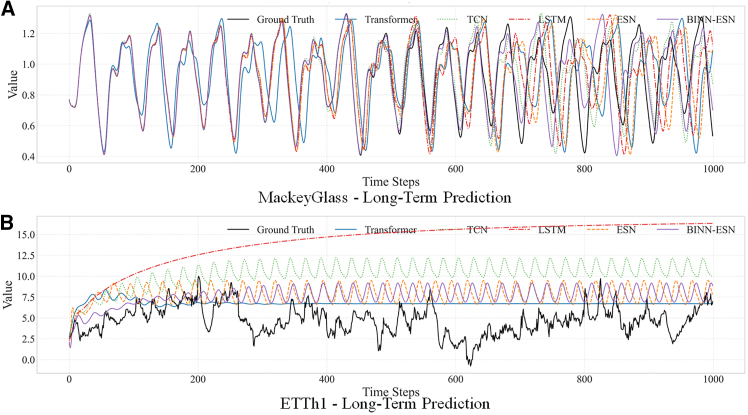
Time-series comparison for Mackey-Glass and ETTh1 from 1000-step free-running predictions Ground truth is shown in black.

To complement the quantitative error metrics, we examine whether each model has genuinely learned the underlying dynamic structure of the target system by inspecting the phase-space trajectories reconstructed from 2000-step free-running predictions. The compact 3D attractor overview (Figure 11) provides a holistic geometric comparison, while the dimension-wise trajectories (Figure 12) and 1D time series (Figure 13) reveal the per-variable temporal fidelity that is not always apparent in the 3D projection.

A more detailed dimension-wise analysis, however, reveals that even visually plausible 3D trajectories can conceal substantial per-component deviation. On the Lorenz system (Figure 12A), the x-axis trajectory confirms that BINN-ESN tracks the ground truth faithfully across the entire horizon; TCN exhibits a clear amplitude offset from the very first step, and both LSTM and ESN begin to diverge gradually after approximately 200 steps, consistent with their lower VPT on this system. On the y- and z-axes, the same hierarchy persists: BINN-ESN remains closest to the ground truth, while TCN and Transformer deviate most severely from the start.

The Chen system (Figure 12B) presents a contrasting pattern. Although all models are visually close in the early phase-space trajectory, the x-axis reveals that TCN and Transformer begin to deviate at approximately 100 steps, whereas LSTM and ESN maintain reasonable alignment for longer. The z axis is notable in that LSTM slightly outperforms ESN and approaches the BINN-ESN level of fidelity. On the Lü system (Figure 12C), all models deviate from the ground truth even at the first step on the X- and Y axes, although they broadly preserve the oscillatory waveform morphology; on the z axis, LSTM achieves the best individual-component agreement.

The Mackey-Glass system (Figure 13) provides the clearest separation among models: BINN-ESN adheres to the ground-truth trajectory with minimal deviation throughout the entire 1000-step horizon, while LSTM and ESN begin to drift noticeably at approximately 260 steps and Transformer at approximately 80 steps. For ETTh1 (Figure 13), all models produce near-flat trajectories, a hallmark of complete learning failure that is consistent with the flat ACF patterns observed in the preceding section.

##### Dynamic fingerprinting via recurrence plots

**Figure 14:**
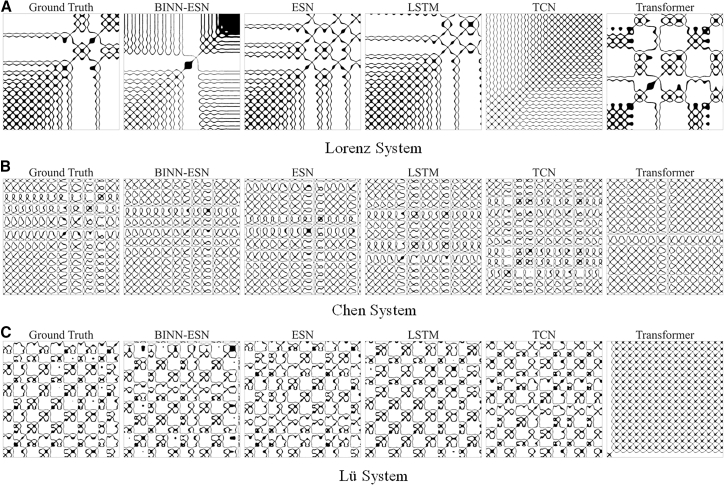
Dynamic structure comparison via recurrence plots across five systems In each row, panels correspond to ground truth, BINN-ESN, ESN, LSTM, and Transformer (left to right). Similarity to the ground-truth RP indicates better preservation of the system’s intrinsic phase-space recurrence structure.

**Figure 15:**
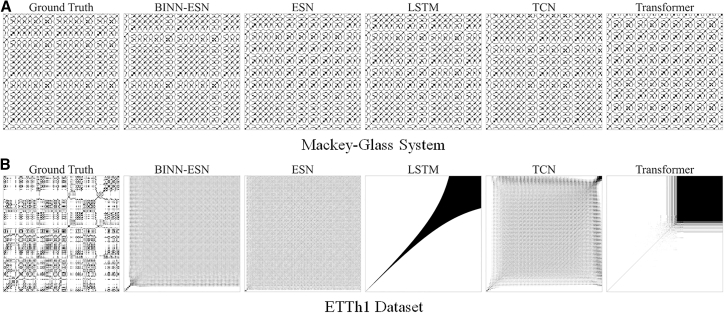
Recurrence plots for Mackey-Glass and ETTh1 See caption of Figure 14 for panel ordering convention.

RPs provide a complementary structural fingerprint by visualizing the times at which a trajectory revisits similar states in phase space. The RP panels for all five systems are shown in Figures 14 and 15.

On the Lorenz system (Figure 14A), the BINN-ESN plot preserves the characteristic diagonal line structures and the overall checkered texture of the ground truth. ESN retains a partial structure but with notably interrupted lines. LSTM produces a sparse plot with fewer recurrence points than the ground truth. Transformer shows a largely dark plot with few visible recurrence structures. The Chen system (Figure 14B) exhibits a broadly similar pattern across models, with BINN-ESN most closely following the ground truth, ESN showing moderate degradation, and LSTM and Transformer showing reduced recurrence structure. On the Lü system (Figure 14C), ESN shows more pronounced degradation relative to the other chaotic systems, while LSTM and Transformer again display sparse recurrence patterns.

The Mackey-Glass system (Figure 15A) further illustrates these differences: Its ground-truth RP displays thick, continuous diagonal bands characteristic of delay-feedback systems, which BINN-ESN reproduces with the highest fidelity among all models. ESN captures the banded topology but with broken segments. LSTM shows fragmented bands with extended dark regions. Transformer again produces a largely dark plot. On ETTh1 (Figure 15B), the ground-truth RP exhibits a complex multi-scale structure. BINN-ESN retains more of this structural complexity compared to ESN, LSTM, and Transformer, all of which produce substantially sparser patterns on this dataset.

### Computational efficiency analysis

#### Execution time analysis

As computational cost is a critical factor in practical deployment, we now analyze the efficiency of all models under a unified evaluation protocol. The total execution time for 10 independent trials across the five benchmark systems is decomposed into two components: (1) **Search Time**, consumed by Bayesian hyperparameter optimization; and (2) **Training Time**, the actual model training per trial including forward passes, state harvesting, and readout weight optimization (Ridge Regression for ESN-family; BPTT for deep learning models).

For ESN-family models (BINN-ESN and standard ESN), the training time is negligible (16–21 s per trial) compared to search time (794–888 s per trial), owing to the closed-form Ridge Regression. For deep learning baselines (LSTM, TCN, Transformer), the training time is substantial (518–6349 s per trial) and dominates the total cost.

The long-term prediction experiments reuse the optimized model parameters from short-term experiments; thus, the long-term execution time consists only of forward prediction, approximately equal to the search time per trial. No additional training or hyperparameter search is performed.

Across the five-system benchmark, BINN-ESN requires approximately the same order of search time as standard ESN (both ∼800–900 s per trial), yet achieves substantially better long-term prediction stability (as shown in Table 2). Compared with gradient-trained deep baselines, BINN-ESN is 2–14× more efficient in total execution time, primarily due to its lightweight training phase. The long-term prediction experiments, which reuse the optimized parameters, incur no additional search cost, making the effective cost per long-term trial approximately equal to the search time shown above. Together with the performance analysis, these results demonstrate that BINN-ESN achieves a favorable trade-off between ESN-family and deep-learning approaches: It retains the computational efficiency of ESN-family models while substantially narrowing the long-term stability gap with recurrent deep-learning baselines.

#### Model memory allocation

**Figure 16:**
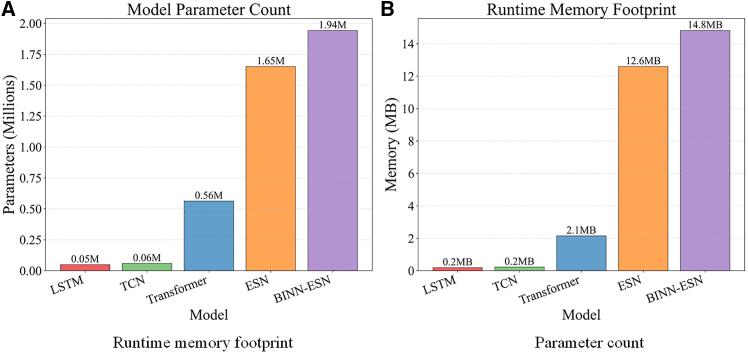
Runtime memory footprint (left) and parameter count (right) comparison across models

Figure 16 and Table 4 report the runtime memory allocation and parameter counts of the optimized models. For ESN-family models (BINN-ESN and standard ESN), memory is dominated by the recurrent weight matrix **W**, which stores the reservoir connectivity. For deep learning models (LSTM, TCN, Transformer), memory includes all trainable parameters and optimizer states. Notably, the ESN-family models require substantially more memory (12.60–14.81 MB) than the deep learning baselines (0.19–2.15 MB). BINN-ESN uses 14.81 MB, approximately 18% more than standard ESN (12.60 MB), which is attributable to its additional brain-inspired neuronal complexity in the MCCNN architecture. This overhead is a direct consequence of the more sophisticated internal state dynamics of the MCCNN neuron and is consistent with the design rationale of the model. Given that the ESN architecture inherently demands a large recurrent weight matrix, the memory premium of BINN-ESN over ESN is modest relative to its substantial gains in long-term stability, making the trade-off acceptable in practical deployment scenarios.

**Table 4:** Runtime, memory allocation, and parameter counts of optimized models

Model	Parameters	Memory (MB)	Ratio vs. BINN-ESN
BINN-ESN	1,941,812	14.81	1.00
Standard ESN	1,651,221	12.60	0.85
Transformer	562,755	2.15	0.15
TCN	58,691	0.22	0.02
LSTM	48,816	0.19	0.01

### Mechanistic insights into the performance of the BINN-ESN

Having established the position of the BINN-ESN as a high-performance yet efficient model, we now turn to a series of diagnostic experiments that are designed to elucidate the underlying mechanisms responsible for its capabilities. The goal is to move beyond *what* the model achieves and understand *why* its brain-inspired design is so effective and why its performance exhibits high variance. We investigate this from three perspectives: the intrinsic potential of a single neuron, the emergent collective behavior of the reservoir, and the causal contribution of its key bio-inspired components.

#### The intrinsic dynamical capacity of an MCCNN neuron

Bearing in mind the foundational hypothesis of this work, that is, endowing reservoir neurons with richer internal dynamics will lead to a more powerful computational substrate, we first analyze the intrinsic capabilities of the proposed MCCNN neurons in isolation. To this end, a single MCCNN neuron is configured with the optimal parameters learned for the Lorenz system (Seed 9) and driven by a canonical sinusoidal stimulus.

**Figure 17:**
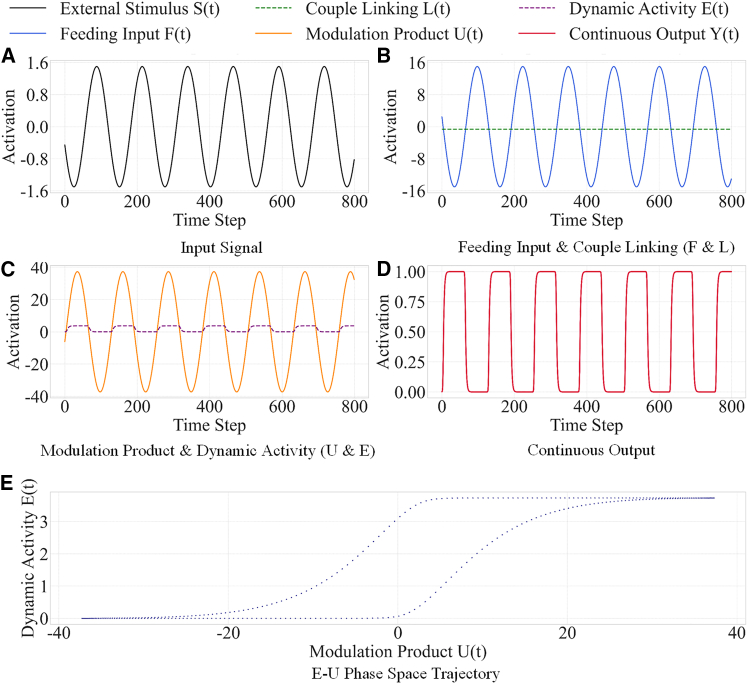
Analysis of the intrinsic dynamics of a single MCCNN neuron under a sinusoidal stimulus using parameters optimized for the Lorenz system (Seed 9) (A) Input signal. (B) Feeding input *F*(*t*) and couple linking *L*(*t*). (C) Modulation product *U*(*t*) and dynamic activity *E*(*t*). (D) Continuous output *Y*(*t*). (E) E-U phase space trajectory.

The results depicted in Figure 17 demonstrate the capacity of the MCCNN to serve as a dynamic transducer. (A) shows the simple sinusoidal input signal. After being processed through the internal state variables of the neuron, this simple input is transformed into a highly nonlinear and complex sequence. The feeding input *F*(*t*) (B) tracks the stimulus closely, while the coupling term *L*(*t*) exhibits subtle oscillatory variations around its baseline. The decisive nonlinear interaction occurs between the modulation product *U*(*t*) and the dynamic activity *E*(*t*) (C): *U*(*t*) is amplified into a series of sharp peaks with large amplitude excursions, while *E*(*t*) exhibits a slower adaptive tracking behavior that broadly follows the stimulus waveform, together producing a dynamic threshold mechanism. This culminates in the final continuous output *Y*(*t*) (D), which exhibits clear bistable switching between the two attractor states (near 0 and near 1) in response to the sinusoidal drive. The E-U phase-space trajectory (E) reveals a diagonal band structure, indicating that the MCCNN neuron operates as a genuine nonlinear dynamical system in which *U* and *E* evolve along correlated but distinct paths.

#### The emergent computational mode and its optimization challenge

Having established the potential of individual neurons, we next investigate the emergent collective behavior of the entire reservoir by analyzing the probability distribution of all the neuron activation values during a representative trial conducted on the Lorenz system.

**Figure 18:**
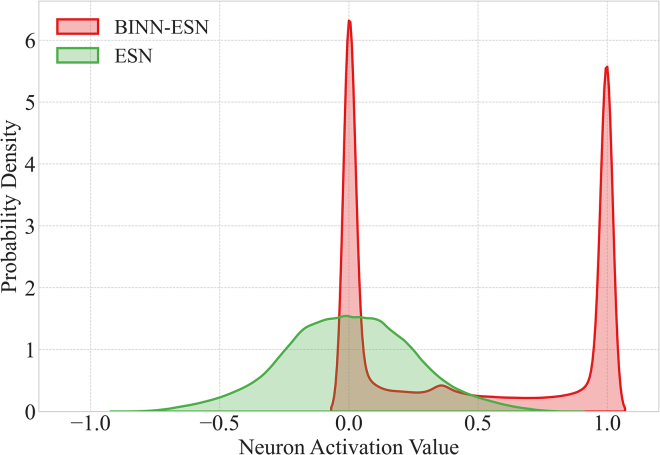
Probability density distributions of the reservoir activation states (specifically, the neuronal output *Y*) observed for the BINN-ESN and standard ESN during the prediction task on the Lorenz system ESN (green) shows a broad unimodal distribution centered around 0.5, tapering symmetrically toward both extremes. BINN-ESN (purple) exhibits a sharply bimodal distribution with peaks at the two attractor states (near 0 and 1).

Significant differences between the computational strategies are evident in Figure 18. The standard ESN produces a broad distribution centered around 0.5, with density tapering symmetrically toward both extremes, resembling a unimodal analog spread. In contrast, the BINN-ESN self-organizes into a sharply bimodal distribution with activation peaks at the two attractor states (near 0 and near 1), corresponding to the binary operating regime of the MCCNN neurons. This bistable “digital switch” organization provides the reservoir with stable reference states that are robust to small perturbations, which we hypothesize contributes to the improved long-term stability of BINN-ESN in free-running prediction.

The large performance variance across the 10 independent trials (reflected in the standard deviations of VPT and MSE) indicates a multi-source effect: sensitivity of the bistable activation regime to hyperparameter selection, combined with trial-wise independence in trajectory initialization, Bayesian search paths, and model initialization. The dynamic 13-dimensional parametric space of the MCCNN endows the BINN-ESN with superior performance potential compared to the ESN, but also introduces a more complex optimization landscape. The Bayesian optimization protocol does not guarantee convergence to the best-performing activation regime in every trial, consistent with the observed cross-seed variability.

#### Deconstructing the achieved performance via an ablation study

To causally link the bio-inspired components of the MCCNN to its performance potential and to further examine performance trade-offs under the revised protocol, we conducted an updated ablation experiment on the Lorenz system. Two simplified variants were considered: one without dynamic activity (*E*) and one without the coupling mechanism (*β* = 0), and both were compared against the full BINN-ESN and the standard ESN.

**Figure 19:**
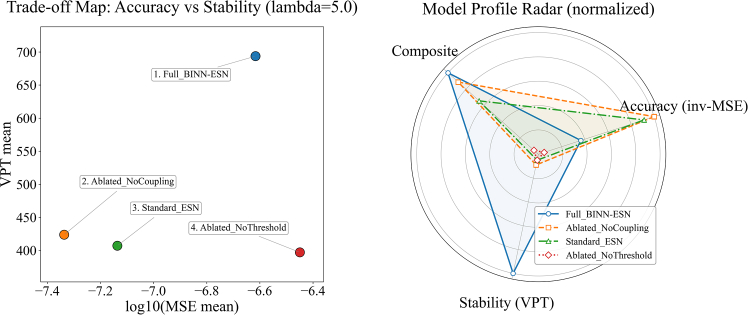
Updated ablation visualization on Lorenz under the revised protocol: combined view of the short-term/long-term trade-off map and the normalized profile of Accuracy (inverse-MSE), Stability (VPT), and Composite performance

In this revised comparison, we jointly evaluate short-term precision and long-term stability through a composite score,

(Equation 5) $$J=\log\left(MSE+\epsilon \right)+\lambda \cdot \max\left(0,1-\frac{VPT}{{VPT}_{\max}}\right),\;\lambda =5.0,$$

where lower values indicate better integrated performance. As shown in Table 5 and Figure 19, the full BINN-ESN achieves the best composite score and the highest mean VPT, while preserving a competitive MSE level. This supports our central claim that the bio-inspired mechanisms are critical for stability-oriented forecasting.

**Table 5:** Updated ablation results on the Lorenz system under the revised protocol (seeds 0–2)

Model/architecture	MSE (mean ± std)	VPT (mean ± std)	Composite score
Full BINN-ESN	2.42e-07 ± 9.03e-08	693.67 ± 186.61	−15.2345
Ablated (w/o coupling)	4.61e-08 ± 3.02e-08	423.67 ± 276.94	−14.9470
Standard ESN (baseline)	7.30e-08 ± 3.56e-08	407.00 ± 264.97	−14.3659
Ablated (w/o threshold)	3.55e-07 ± 2.35e-07	397.00 ± 87.94	−12.7115

Composite score is defined in Equation 5 with λ = 5.0 (lower is better).

### Hyperparameter sensitivity analysis

To address Reviewer 3’s concern regarding the robustness of our model to its optimized parameters, we conduct a comprehensive hyperparameter sensitivity analysis on six key MCCNN/ESN parameters: *α*_*f*_, *α*_*l*_, *α*_*e*_, *β*, *ρ* (spectral radius), and *V*_*F*_. Using the optimized BINN-ESN configuration for the Lorenz system (seed = 0 fixed), we vary one parameter at a time across its search range while fixing all others at their optimal values. These six parameters were selected because they directly govern the MCCNN’s multi-timescale dynamics (*α*_*f*_, *α*_*l*_, *α*_*e*_), nonlinearity (*β*), reservoir stability (*ρ*), and synaptic gain (*V*_*F*_).

**Figure 20:**
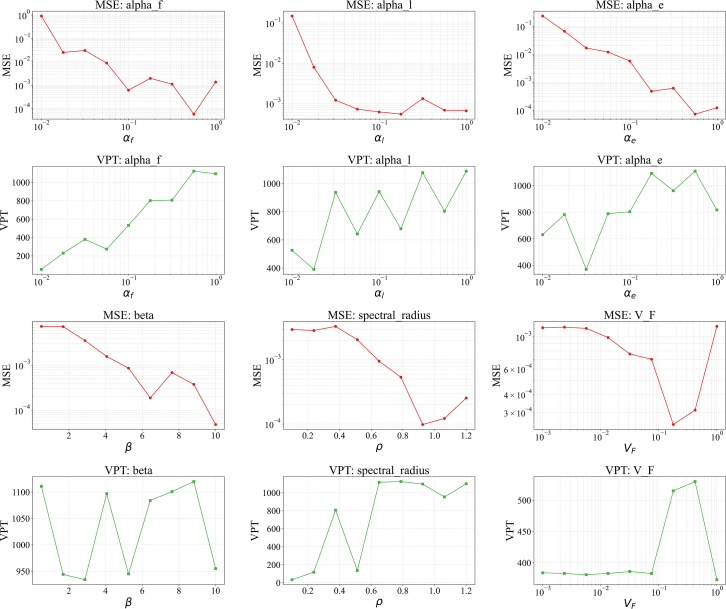
Comprehensive hyperparameter sensitivity analysis for six key MCCNN/ESN parameters on the Lorenz system Top row: MSE (log-scale) vs. parameter value. Bottom row: VPT (steps) vs. parameter value.

Figure 20 presents the results. Key findings: (1) *α*_*f*_ shows strong sensitivity with MSE varying over 4 orders of magnitude; (2) *α*_*e*_ exhibits a clear optimal window (0.31–0.99) where MSE reaches ∼ 4 × 10^−4^ and VPT exceeds 500 steps; (3) *β* and spectral radius both favor larger values, confirming the importance of nonlinear dynamics and reservoir stability; (4) VPT peaks at specific parameter configurations, demonstrating that long-term prediction stability is not merely a result of smoothing but requires proper dynamic balance. Crucially, the searched optimal parameters (e.g., *α*_*f*_ = 0.426, *α*_*e*_ = 0.605, *β* = 9.79) lie within or near these optimal regions, validating the Bayesian optimization results. Parameters causing extreme dynamics (e.g., very small *α*_*f*_) produce poor VPT, confirming gains stem from genuine dynamic modeling rather than noise suppression.

The tested ranges, optimized values, and identified effective regions are summarized in Table 6.

**Table 6:** Hyperparameter sensitivity analysis configuration and results for the Lorenz system

Parameter name	Symbol	Optimal value	Tested range	Optimal range
Feeding input exponential decay	*α*_*f*_	0.426	[0.01, 0.99]	0.17–0.56
Couple linking exponential decay rate	*α*_*l*_	0.711	[0.01, 0.99]	∼0.10
Dynamic activity threshold decay	*α*_*e*_	0.605	[0.01, 0.99]	0.31–0.99
Linking strength	*β*	9.795	[0.5, 10.0]	8.0–10.0
Spectral radius	*ρ*(**W**)	1.027	[0.1, 1.2]	0.79–1.20
Feeding pathway weighting factor	*V*_*F*_	0.632	[0.001, 1.0]	0.18–0.42

Optimal values are obtained from Bayesian optimization (seed = 0).

## Discussion

In this work, a RC framework (BINN-ESN) designed to bridge the gap between the computational efficiency of traditional ESNs and the high performance of complex deep learning models, such as LSTM networks, was proposed. The core of our contribution lies in the introduction of the MCCNN neuron, which is a sophisticated dynamical unit whose design principles are inspired by the adaptive and integrative properties of biological neurons.

Through a comprehensive and statistically grounded set of experiments, we find that BINN-ESN offers a practical balance between performance and efficiency. In our benchmarks, deep learning models are generally stronger in short-term accuracy, whereas BINN-ESN can provide long-term advantages on key chaotic systems and requires less total computation than the GPU-accelerated deep learning baselines (LSTM, TCN, and Transformer) under the present evaluation protocol. Our analysis suggests that this behavior is associated with the bio-inspired design of the network. MCCNN components related to neuronal adaptation and dendritic-like integration^8^^,^^9^ may support the emergence of a “digital switch” computational mode that can mitigate error accumulation in chaotic forecasting.

In addition, our findings support exploring brain-inspired dynamics in computational models and indicate that specific bio-inspired mechanisms may enhance robustness and contribute to efficient and interpretable solutions for complex time-series prediction.

### Limitations of the study

Although the study reveals a compelling balance between performance and efficiency for the BINN-ESN, it also identifies its limitations and suggests areas for future research.

1. The introduction of the MCCNN neuron expands the hyperparameter space to 13 dimensions, creating a complex optimization landscape. Our analysis indicates that the model’s superior performance relies on converging to a specific bimodal activation state. However, the convergence to this state exhibits variance across trials when using standard Bayesian optimization. Although the overall execution cost remains favorable (2–14× lower total time than deep learning baselines under our protocol, as shown in Table 3), the optimization stage still requires robust initialization strategies. Future work will investigate evolutionary algorithms guided by Lyapunov exponents to stabilize the convergence process.

**Table 3:** Computational efficiency comparison

Model	Search time (s)	Training time (s)	Total (10 trials, s)
BINN-ESN	888.1 ± 176.9	20.7 ± 13.2	45437.64
Standard ESN	793.8 ± 132.3	16.4 ± 13.2	40506.35
LSTM (GPU)	2025.4 ± 412.9	517.6 ± 444.3	127153.76
TCN	12058.0 ± 2330.5	6348.6 ± 1798.7	920328.83
Transformer	1139.7 ± 269.5	641.1 ± 359.2	89040.64

The values represent the execution time (in seconds) per trial, averaged over 10 trials across five systems (Lorenz, Chen, Lü, Mackey-Glass, ETTh1). The time is decomposed into search time (Bayesian hyperparameter optimization) and training time (model training per trial). The total time for 10 trials is also provided.
2. The bimodal “digital switch” behavior observed in the reservoir is currently interpreted through phenomenological analysis. A formal theoretical framework is required to fully explain the conditions under which this regime emerges. Future studies could employ random dynamical systems theory and bifurcation analysis to provide a rigorous mathematical description of the reservoir’s stability properties and its transition to the bimodal state.
3. The current study has included an initial real-world benchmark (ETTh1) in addition to synthetic chaotic systems, but broad cross-domain validation is still limited. While this provides first evidence under noisy, non-stationary conditions, more diverse empirical datasets from finance, meteorology, and neuroscience are required to fully establish practical generalizability.

## Resource availability

### Lead contact

Further information and requests for code and data should be directed to and will be fulfilled by the Lead Contact, Prof. Yide Ma (ydma@lzu.edu.cn).

### Materials availability

Not applicable.

### Data and code availability

• All data used in the paper were generated by ordinary differential equations described in Sec.10.3.1.
• The code is available on https://github.com/Jizhao-Liu/code-for-BINN-ESN.
• Any additional information required to reanalyze the data reported in this paper is available from the lead contact upon request.

## Acknowledgments

L.M. gratefully acknowledges the support received from the Hundred Talents program of the 10.13039/501100005408University of Electronic Science and Technology of China, the Outstanding Young Talents Program (Overseas) program of the 10.13039/501100001809National Natural Science Foundation of China, and the talent programs of the Sichuan province and Chengdu municipality. Some experiments are supported by the Supercomputing Center of Lanzhou University.

## Author contributions

P.M. and X.W. conceived the presented idea. P.M., X.W., and H.Q. carried out the experiment. J.L. and L.M. developed the theory. P.M. and X.W. took the lead in writing the manuscript. P.M., X.W., and Y.M. revised the manuscript. Y.M. supervised the findings of this work. P.M. and Y.M. provided the financial support.

## Declaration of interests

The authors declare that they have no known competing financial interests or personal relationships that could have appeared to influence the work reported in this paper.

## STAR★Methods

### Key resources table

**Table:** 

REAGENT or RESOURCE	SOURCE	IDENTIFIER
**Software and algorithms**		

Published on public websites	https://github.com/Jizhao-Liu/code-for-BINN-ESN	github.com

### Experimental model and study participant details

Omitted as our study does not involve biological models.

### Method details

In this section, the theoretical and architectural foundations of our work are presented. First of all, the overall architecture of the proposed BINN-ESN is presented. Thereafter, its operational principles within the reservoir computing paradigm are established, and the MCCNN approach is presented.

#### The Brain-Inspired Neuron Network-Based echo state network

The BINN-ESN is a recurrent neural network that integrates complex brain-inspired neuronal dynamics into the framework of reservoir computing. Its architecture conforms to the canonical three-layer structure of an ESN consisting of an input layer, a recurrent reservoir, and a linear output layer (Below Figure).

**Figure 21:**
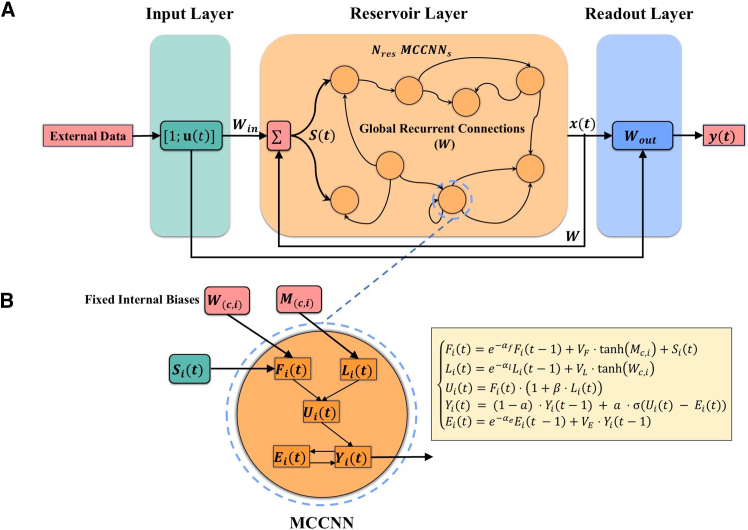
The BINN-ESN framework (A) High-level architecture, showing the input layer, the reservoir layer with global recurrent connections (**W**), and the readout layer. (B) A detailed view of a single MCCNN neuron within the reservoir. Its five internal state variables, the influences of the input drive *S*_*i*_(*t*) and the fixed internal biases (*M*_*c*,*i*_, *W*_*c*,*i*_), and its mathematical formulation are illustrated.

##### Network components and connectivity

The static connectivity of the BINN-ESN is defined by three weight matrices. The input weight matrix, $W_{\text{in}}\in R^{N_{\text{res}}\times \left(1+N_{\text{in}}\right)}$, connects the external input signal to the reservoir. The recurrent weight matrix, $W\in R^{N_{\text{res}}\times N_{\text{res}}}$, defines the internal topology of the reservoir. Both matrices are randomly initialized and remain fixed after their initialization, with the spectral radius of a sparse **W** typically scaled close to unity to ensure the echo state property.^18^ The output weight matrix, $W_{\text{out}}\in R^{N_{\text{out}}\times \left(1+N_{\text{in}}+N_{\text{res}}\right)}$, maps the combined system state to the final prediction and is the sole trainable component which is learned via ridge regression.^36^ Specifically, **W**_out_ is computed analytically in closed form. After the washout phase, the reservoir states **h**(*t*) = [1; **u**(*t*); **x**(*t*)] are collected row-wise into a state matrix $H\in R^{T_{\text{train}}\times \left(1+N_{\text{in}}+N_{\text{res}}\right)}$, and the corresponding target vectors are stacked into **Y**_target_. The output weights are then computed as:

(Equation 6) $$W_{\text{out}}=Y_{\text{target}}^{⊤}H{\left(H^{⊤}H+\lambda I\right)}^{-1}$$

where *λ* is the Tikhonov regularization coefficient that prevents overfitting by penalizing large weight norms. This single-shot, gradient-free closed-form solution is what enables the BINN-ESN’s remarkable computational efficiency.

##### Computational flow

At each discrete time step *t*, the computational flow of the BINN-ESN proceeds as follows. First of all, the reservoir state acquired from the previous time step **x**(*t* − 1), and the current external input **u**(*t*), are used to compute a total affine input drive $S\left(t\right)\in R^{N_{\text{res}}}$ for all reservoir neurons:

(Equation 7) $$S\left(t\right)=W_{\text{in}}\left[1;u\left(t\right)\right]+Wx\left(t-1\right).$$

This input is then processed by the internal dynamics of the core computational units of the reservoir (the MCCNN neurons), leading to the collective output vectors derived from these neurons **Y**(*t*) that form the new state of the reservoir for the current time step:

(Equation 8) x(t)=Y(t)

Finally, the network prediction **y**(*t*) is generated by the linear readout layer using this newly computed state:

(Equation 9) $$y\left(t\right)=W_{\text{out}}\left[1;u\left(t\right);x\left(t\right)\right]$$

The specific dynamics governing the transformation from **S**(*t*) and **x**(*t* − 1) to the new state **x**(*t*) is encapsulated within the MCCNN neurons. This is detailed next as the central contribution of our framework.

#### The modified continuous coupled neural network (MCCNN)

The design of the MCCNN neurons is a significant adaptation of the CCNN^9^ with the aim of emulating the key computational principles of biological neurons in a generalized and efficient manner. Their five internal state variables are inspired by distinct neuronal processes.The mammalian primary visual cortex was specifically selected as the biological inspiration due to its innate capability to process continuous, complex, and noisy spatiotemporal signal streams without suffering from runaway excitation. As foundational studies on visual cortex-inspired models (such as PCNN and CCNN) have demonstrated, neurons in this region employ sophisticated nonlinear integration and dynamic refractory periods to maintain a stable “edge of chaos” state, efficiently filtering redundant noise while remaining highly sensitive to transient dynamic shifts.^9^^,^^30^ By abstracting these specific biological mechanisms into the MCCNN architecture, we provide the artificial reservoir with a natural defense against exponential error accumulation—a critical necessity for achieving long-term stability in chaotic time-series forecasting.

The *feeding input* (*F*_*i*_) and *couple linking* (*L*_*i*_) variables are, for instance, designed to model the principle of dendritic integration (the process by which a neuron integrates signals from numerous synaptic sources onto its dendritic tree). The *modulation product* (*U*_*i*_) multiplies the F and L pathways instead of realizing a simple linear summation, allowing complex and nonlinear synaptic interactions (such as shunting effects) to be reproduced in a manner similar to those observed in biological systems.^37^ The *dynamic activity* (*E*_*i*_) modulated by the neuron’s own past outputs directly corresponds to the neuron’s adaptation and refractory phenomena. Such a negative feedback loop is essential for preventing uncontrolled excitation and for making a neuron sensitive to temporal changes in its input; a feature which is indispensable for generating complex temporal patterns.

To transform this bioinspired concept into a versatile computing unit, we introduce three main modifications to the original CCNN formulation.

##### The foundational CCNN model and its limitations

The original CCNN model describes the state of a neuron (*i*, *j*) via five coupled difference equations:

(Equation 10) $$\left\{\begin{array}{cc} F_{ij}\left(t\right) & =e^{-\alpha_{f}}F_{ij}\left(t-1\right)+V_{F}\underset{k,l}{\sum }M_{\mathrm{ijkl}}Y_{kl}\left(t-1\right)+S_{ij}\left(t\right)\\ L_{ij}\left(t\right) & =e^{-\alpha_{l}}L_{ij}\left(t-1\right)+V_{L}\underset{k,l}{\sum }W_{\mathrm{ijkl}}Y_{kl}\left(t-1\right)\\ U_{ij}\left(t\right) & =e^{-\alpha_{f}}U_{ij}\left(t-1\right)+F_{ij}\left(t\right)\left(1+\beta L_{ij}\left(t\right)\right)\\ Y_{ij}\left(t\right) & =\frac{1}{1+e^{-\left(U_{ij}\left(t\right)-E_{ij}\left(t\right)\right)}}\\ E_{ij}\left(t\right) & =e^{-\alpha_{e}}E_{ij}\left(t-1\right)+V_{E}Y_{ij}\left(t-1\right) \end{array}\right)$$

In this formulation, the interneuronal connectivity is defined by the rank-4 weight tensors *M*_*ijkl*_ and *W*_*ijkl*_. To capture local interactions between pixels for images processing applications, these tensors are generally defined manually using spatially structured kernels, such as Gaussian or Laplacian filters. What particularly motivated our modifications is the observation that the initial CCNN is handcrafted and oriented towards specific applications, and is therefore unsuitable for general-purpose time series forecasting tasks where such spatial priors do not exist.

##### Key modifications and the final MCCNN formulation

Architectural Adaptations

1. In the proposed model, the rank-4 weight tensors (*M*_*ijkl*_, *W*_*ijkl*_) that defined the spatial coupling in the original CCNN are replaced by scalar biases (*M*_*c*,*i*_, *W*_*c*,*i*_) specific to each neuron and randomly initialized and transformed by a tanh function. This major change allows the replacement of the computationally expensive interneuronal weighted sum by a fixed internal drive, thereby making each neuron independent of the network structure and capable of functioning as a standalone computing unit. Correspondingly, the state of the neuron is now described by a single index *i*, rather than the spatial grid indices (*i*, *j*) used in the original CCNN. These scalar biases are initialized for each neuron by drawing from a uniform distribution *U*(−1, 1).
2. The independent recursive decay term for the modulation product *U*_*i*_ (i.e., the term $e^{-\alpha_{f}}U_{ij}\left(t-1\right)$ in the original CCNN) is removed and is updated in a direct memoryless function of the current states of *F*_*i*_ and *L*_*i*_. Such a simplification reduces complexity of the internal state of the model and mitigates the risk of overfitting by preventing the accumulation of redundant state information.
3. The computation of neuron output *Y*_*i*_(*t*), which incorporates a leaky integrator mechanism is a weighted combination of its past state *Y*_*i*_(*t* − 1) and the current activation governed by a leak rate hyperparameter *a*. This enhances the temporal processing capabilities of the neuron and its robustness to high-frequency noise; an essential feature for handling continuous time series data.

##### Final mathematical formulation

Applying the three architectural adaptations described above to the CCNN model (Equation 10) yields the final mathematical formulation of our MCCNN neuron. Given its input drive *S*_*i*_(*t*), the MCCNN variables are updated sequentially to produce the final output *Y*_*i*_(*t*). This computational process follows a specific, recurrent information flow, as conceptually illustrated in Below Figure. The input drive first updates the feeding and linking states, which are then nonlinearly combined. The obtained result is then compared with the adaptive dynamic activity state to produce the final output, which in turn modulates the future dynamic activity and forms a feedback loop.

The precise mathematical formulation of these dynamics is as follows:

**Figure 22:**
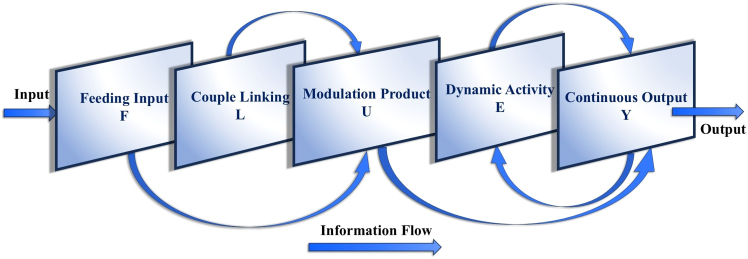
Conceptual outlook of the information flow and computational stages within the MCCNN neuron The process involves a feedforward cascade from the input to the output, together with multiple internal feedback loops that induce the rich history-dependent dynamics of the neuron.

(Equation 11) $$\left\{\begin{array}{cc} F_{i}\left(t\right) & =e^{-\alpha_{f}}F_{i}\left(t-1\right)+V_{F}\cdot \tanh\left(M_{c,i}\right)+S_{i}\left(t\right)\\ L_{i}\left(t\right) & =e^{-\alpha_{l}}L_{i}\left(t-1\right)+V_{L}\cdot \tanh\left(W_{c,i}\right)\\ U_{i}\left(t\right) & =F_{i}\left(t\right)\cdot \left(1+\beta \cdot L_{i}\left(t\right)\right)\\ E_{i}\left(t\right) & =e^{-\alpha_{e}}E_{i}\left(t-1\right)+V_{E}\cdot Y_{i}\left(t-1\right)\\ Y_{i}\left(t\right) & =\left(1-a\right)\cdot Y_{i}\left(t-1\right)+a\cdot \sigma \left(U_{i}\left(t\right)-E_{i}\left(t\right)\right) \end{array}\right)$$

where all the variables except *S*_*i*_ are internal to the corresponding neuron. Unlike the fixed kernels of the original CCNN, all key dynamical parameters in the MCCNN are treated as hyperparameters and optimized for the specific task at hand.

##### Feeding input *F*

The feeding input *F*_*i*_ integrates the total affine input drive *S*_*i*_(*t*) with a neuron-specific internal bias. This process models the primary dendritic signal aggregation step and is governed by the following update rule:

(Equation 12) $$F_{i}\left(t\right)=e^{-\alpha_{f}}F_{i}\left(t-1\right)+V_{F}\cdot \tanh\left(M_{c,i}\right)+S_{i}\left(t\right).$$

Here, $e^{-\alpha_{f}}$ is the exponential decay term that controls how much of the previous state is retained. *M*_*c*,*i*_ represents the randomly initialized, fixed internal bias for the feeding pathway. Its contribution is scaled by the weighting factor *V*_*F*_. The tanh function constrains the bias to prevent runaway activation.

##### Couple linking *L*

The couple linking state *L*_*i*_ provides a secondary modulatory pathway that is analogous to shunt inhibition or other nonlinear dendritic interactions. It is driven by its own internal bias and evolves according to the following equation:

(Equation 13) $$L_{i}\left(t\right)=e^{-\alpha_{l}}L_{i}\left(t-1\right)+V_{L}\cdot \tanh\left(W_{c,i}\right).$$

Similar to the feeding input, this state has an exponential decay rate of $e^{-\alpha_{l}}$ and an internal bias of *W*_*c*,*i*_ that are scaled by a weighting factor *V*_*L*_. The primary role of this component is to interact with the feeding input to create more complex dynamics.

##### Modulation product *U*

The modulation product *U*_*i*_ represents the core nonlinear interaction within the neuron. It combines the feeding input *F*_*i*_ and the couple linking term *L*_*i*_, allowing for complex gain modulation:

(Equation 14) $$U_{i}\left(t\right)=F_{i}\left(t\right)\cdot \left(1+\beta \cdot L_{i}\left(t\right)\right).$$

The hyperparameter *β* is the linking strength that dictates the intensity of the modulation effect.

##### Dynamic activity *E*

The dynamic activity *E*_*i*_ acts as an adaptive threshold, mimicking the refractory period and adaptation phenomena observed in biological neurons. Its value is modulated by the past output of the neuron itself, creating the negative feedback loop:

(Equation 15) $$E_{i}\left(t\right)=e^{-\alpha_{e}}E_{i}\left(t-1\right)+V_{E}\cdot Y_{i}\left(t-1\right).$$

The threshold decays over time with the factor $e^{-\alpha_{e}}$ and increases proportionally to the previous output *Y*_*i*_(*t* − 1), scaled by the threshold factor *V*_*E*_. This mechanism makes the neuron sensitive to input changes and prevents sustained firing.

##### Continuous output *Y*

The final output *Y*_*i*_ computed via a leaky integrator mechanism, combines the previous output of the neuron with the new activation as follows:

(Equation 16) $$Y_{i}\left(t\right)=\left(1-a\right)\cdot Y_{i}\left(t-1\right)+a\cdot \sigma \left(U_{i}\left(t\right)-E_{i}\left(t\right)\right).$$

The leakage rate *a* balances the influences of the memory of the neuron (its past state) and its current input-driven activation, the sigmoid function *σ* ensures that the output is bounded, and the term *U*_*i*_(*t*) − *E*_*i*_(*t*) signifies that the neuron “fires” only when the modulated input *U*_*i*_ exceeds the adaptive threshold *E*_*i*_.

#### Algorithm pipeline and echo state property

To provide full transparency on the training and inference procedure, we present the complete algorithmic pipeline in Algorithm 1. All MCCNN internal states (*F*_*i*_, *L*_*i*_, *E*_*i*_, *Y*_*i*_) are strictly initialized to zero to prevent arbitrary biases from influencing the network dynamics.

Algorithm 1 **BINN-ESN Training and Inference Pipeline**

**Require:** Training data ${\left(u\left(t\right),y_{\text{target}}\left(t\right)\right)}_{t=1}^{T}$; hyperparameters *θ* = {*N*_res_, *ρ*, *α*_*f*_, *α*_*l*_, *α*_*e*_, *β*, *V*_*F*_, *V*_*L*_, *V*_*E*_, *a*, *λ*}; washout length *T*_0_

**Ensure:** Trained output weights **W**_out_

 
**— Phase 1: Initialization —**

1: Generate **W**_in_ (random, uniform), **W** (sparse, spectral radius → *ρ*)

2: Generate fixed internal biases $M_{c,i},W_{c,i}∼U\left[-1,1\right]$

3: Initialize all MCCNN states: *F*_*i*_, *L*_*i*_, *E*_*i*_, *Y*_*i*_ ← 0 *∀i* ∈ {1, *…*, *N*_res_}

 
**— Phase 2: State Harvesting (Training) —**

4: **for** *t* = 1 to *T* **do**

5:   Compute drive: **S**(*t*) ←**W**_in_[1; **u**(*t*)] + **WY**(*t* − 1)

6:   **for** each neuron *i* = 1 to *N*_res_ **do**

7:    $F_{i}\left(t\right)\leftarrow e^{-\alpha_{f}}F_{i}\left(t-1\right)+V_{F}\tanh\left(M_{c,i}\right)+S_{i}\left(t\right)$

8:    $L_{i}\left(t\right)\leftarrow e^{-\alpha_{l}}L_{i}\left(t-1\right)+V_{L}\tanh\left(W_{c,i}\right)$

9:    *U*_*i*_(*t*) ← *F*_*i*_(*t*) ・ (1 + *β* ・ *L*_*i*_(*t*))

10:    $E_{i}\left(t\right)\leftarrow e^{-\alpha_{e}}E_{i}\left(t-1\right)+V_{E}\cdot Y_{i}\left(t-1\right)$

11:    *Y*_*i*_(*t*) ← (1 − *a*)*Y*_*i*_(*t* − 1) + *a* ・ *σ*(*U*_*i*_(*t*) − *E*_*i*_(*t*))

12:   **end for**

13:   **if** *t* > *T*_0_ **then**

14:    Store **h**(*t*) = [1; **u**(*t*); **Y**(*t*)] in state matrix **H**

15:   **end if**

16: **end for**

 
**— Phase 3: Readout Training (Ridge Regression) —**

17: $W_{\text{out}}\leftarrow Y_{\text{target}}^{⊤}H{\left(H^{⊤}H+\lambda I\right)}^{-1}$ ⊳ Equation 6

 
**— Phase 4: Inference (Free-Running Prediction) —**

18: **for** *t* = *T* + 1 to *T* + *T*_pred_ **do**

19:   Use **y**(*t* − 1) as new input **u**(*t*)

20:   Update all MCCNN states (Steps 7–12)

21:   **y**(*t*) ←**W**_out_[1; **u**(*t*); **Y**(*t*)]

22: **end for**

##### Echo state property (ESP) guarantee

A fundamental requirement for any ESN-based architecture is the preservation of the Echo State Property, which ensures that the reservoir state depends solely on the input history and is independent of initial conditions.^18^ Despite the added bio-inspired complexity, the BINN-ESN preserves the ESP through three mechanisms: (1) the exponential decay factors $\left(e^{-\alpha_{f}},e^{-\alpha_{l}},e^{-\alpha_{e}}\right)$ ensure that each internal state variable is a contraction mapping, guaranteeing that transient effects decay over time; (2) the bounded sigmoid activation *σ*(・) ∈ (0, 1) combined with the leaky integration ensures that the output *Y*_*i*_ remains bounded; (3) the spectral radius of **W** is scaled to control the contractivity of the recurrent dynamics. Together, these ensure that the overall state update mapping remains a contraction, satisfying the ESP.

#### Computational complexity and parameter analysis

To rigorously substantiate the efficiency claims regarding the BINN-ESN, we present a theoretical analysis of computational complexity and parameter counts compared to deep learning baselines (e.g., LSTM).

##### Parameter count

The LSTM network possesses a massive trainable parameter space. For a single-layer LSTM with *N*_*h*_ hidden units and *N*_*in*_ input features, the number of trainable parameters is *P*_*LSTM*_ = 4 × ((*N*_*in*_ + *N*_*h*_) × *N*_*h*_ + *N*_*h*_). In contrast, the BINN-ESN strictly adheres to the reservoir computing paradigm. The MCCNN dynamic parameters (*V*_*F*_, *α*_*f*_, *β*, etc.) are global *fixed hyperparameters* determined during the Bayesian search, adding O(1) to the structural complexity. The internal biases (*M*_*c*,*i*_, *W*_*c*,*i*_) are randomly generated and frozen. Therefore, the only trainable parameters are in the readout layer: *P*_*BINN*_ = *N*_*out*_ × (1 + *N*_*in*_ + *N*_*res*_), which is updated purely linearly.

##### Computational complexity

The computational efficiency referred to in this work primarily concerns the *training cost*. An LSTM utilizes Backpropagation Through Time (BPTT), which has a time complexity of $O\left(E\cdot T\cdot N_{h}^{2}\right)$ per trial, where *E* is the number of training epochs and *T* is the sequence length. BPTT requires storing intermediate gradients across the entire temporal unrolling, leading to severe computational overhead.

Conversely, the BINN-ESN training involves two lightweight steps: (1) state harvesting, which takes $O\left(T\cdot N_{\mathrm{res}}^{2}\right)$ for the matrix-vector multiplications plus $O\left(T\cdot N_{\mathrm{res}}\right)$ for the MCCNN scalar updates; and (2) Ridge Regression, which computes the pseudo-inverse in $O\left(T\cdot N_{\mathrm{res}}^{2}+N_{\mathrm{res}}^{3}\right)$ time. Crucially, this operation is single-shot (*E* = 1) and gradient-free.

Even when accounting for the full Bayesian optimization (hyperparameter search) wrapper, evaluating the objective function for BINN-ESN (a fast forward pass and a linear matrix inversion) is orders of magnitude faster than repeatedly executing BPTT gradient updates over hundreds of epochs for the LSTM. This theoretical gap directly translates to the empirical efficiency advantage shown in Section computational efficiency analysis.

To provide empirical validation of the memory footprint, Table 7 presents the measured memory allocation for all models using their optimized hyperparameter configurations. BINN-ESN requires 14.81 MB (1,941,812 parameters), which is only 17% more than the standard ESN (12.60 MB). In contrast, deep learning models achieve dramatically smaller memory footprints despite their larger theoretical parameter spaces: LSTM requires only 190.69 KB (48,816 parameters) and TCN requires 229.26 KB (58,691 parameters). The Transformer falls between these at 2.15 MB (562,755 parameters). Notably, both ESN variants require no gradient storage during training, as their readout layers are optimized via a single-shot Ridge Regression, making their effective memory requirements during training comparable to inference.

**Table 7:** Measured memory allocation of optimized models

Model	Parameters	Memory	Ratio vs. BINN-ESN
LSTM	48,816	190.69 KB	1.3%
TCN	58,691	229.26 KB	1.5%
Transformer	562,755	2.15 MB	14.5%
Standard ESN	1,651,221	12.60 MB	85.0%
BINN-ESN	1,941,812	14.81 MB	100%

#### Automated hyperparameter optimization

To navigate the high-dimensional set of hyperparameters of the BINN-ESN and find optimal configurations in a principled and reproducible manner, Bayesian optimization with Gaussian process surrogates was employed.^38^^,^^39^ This automated strategy is applied consistently to all the models examined in this study.

##### Objective function as a proxy for stability

An essential aspect of our methodology is optimizing for long-term predictive stability, which refers to the model’s ability to generate long autonomous predictions without catastrophic error accumulation. While this is our ultimate goal, directly optimizing a model with respect to its multistep prediction error is usually computationally prohibitive and involves navigating an extremely rugged optimization landscape^40^; a well-known challenge encountered when training recurrent networks for long-term dependencies.^40^ Therefore, a common and effective “proxy-based” optimization strategy was adopted. We hypothesize that a model that excels at accurately capturing the local, one-step-ahead dynamics of a system is more likely to have learned the internal representation of the underlying dynamical rules of the system. Such a representation is a necessary prerequisite for achieving long-term stability. Consequently, the objective function L(θ) was defined as the mean squared error (MSE) of the single-step prediction produced on a dedicated validation set with a length of *T*_val_:

(Equation 17) $$L\left(\theta \right)=\frac{1}{T_{\text{val}}}\overset{T_{\text{val}}}{\underset{t=1}{\sum }}{\left\|y_{\text{true}}\left(t\right)-\hat{y}\left(t;\theta \right)\right\|}_{2}^{2}$$

where **y**_true_(*t*) is the ground-truth target vector at time *t* and $\hat{y}\left(t;\theta \right)$ is the prediction generated by the model using the hyperparameter set *θ*. This optimization scheme is performed for a fixed number of iterations, and the resulting optimal parameter set is then evaluated in both short- and long-term prediction tasks.

The optimized hyperparameters of the BINN-ESN model and the baseline models are detailed in Section quantification and statistical analysis (Tables 8, 10, 11, and 12). To balance the computational cost and thoroughness of the search procedure, a fixed number of optimization iterations was set for each trial. Following the revised experimental protocol, 50 Bayesian optimization iterations (*n*_*calls* = 50) are allocated for the computationally efficient ESN and BINN-ESN models (reflecting their larger hyperparameter spaces), while 25 iterations are used for the more resource-intensive LSTM, TCN, and Transformer baselines, ensuring experiments remain tractable. Within each trial/seed, all models are optimized on the same trajectory split to preserve fairness; across different seeds, trajectories and optimization paths are independently generated.

**Table 8:** Hyperparameter optimization space for the BINN-ESN model

Category	Parameter name	Symbol	Optimization range
Structural (ESN backbone)	reservoir size	*N*_res_	integer [700, 1500]
	input scaling	*s*_in_	real [0.1, 2.0]
	sparsity	–	real [0.05, 0.5]
	spectral radius	*ρ*(**W**)	real [0.1, 1.2]
	regularization coeff.	*λ*_reg_	log-uniform [10^−9^, 10^−2^]
Dynamical (MCCNN neuron)	exponential decay factor (F)	*α*_*f*_	log-uniform [0.001, 1.0]
	exponential decay factor (L)	*α*_*l*_	log-uniform [0.001, 1.0]
	exponential decay factor (E)	*α*_*e*_	log-uniform [0.001, 1.0]
	weighting factor (F)	*V*_*F*_	real [0.001, 1.0]
	weighting factor (L)	*V*_*L*_	real [0.001, 1.0]
	threshold factor (E)	*V*_*E*_	real [0.001, 1.0]
	linking strength	*β*	real [1.0, 10.0]
	output leak rate	*a*	real [0.01, 1.0]

**Table 9:** Hyperparameter optimization space for the standard ESN baseline

Parameter name	Symbol	Optimization range
Reservoir size	*N*_res_	integer [700, 1500]
Input scaling	*s*_in_	real [0.1, 2.0]
Sparsity	–	real [0.05, 0.5]
Spectral radius	*ρ*(**W**)	real [0.1, 1.2]
Leak rate	*a*	real [0.01, 1.0]
Regularization coeff.	*λ*_reg_	log-uniform [10^−9^, 10^−2^]

**Table 10:** Hyperparameter optimization space for the LSTM baseline

Parameter name	Symbol	Optimization range
Number of hidden units	*N*_hidden_	integer [64, 256]
Number of LSTM layers	*N*_layers_	integer [1, 4]
Learning rate	*η*	log-uniform [10^−4^, 10^−2^]
Batch size	*B*	integer [32, 128]
Dropout rate	*p*_dropout_	real [0.1, 0.5]
Sequence length	*T*_seq_	integer [20, 100]

**Table 11:** Hyperparameter optimization space for the TCN baseline

Parameter name	Symbol	Optimization range
Base channel width	*N*_ch_	integer [16, 64]
Kernel size	*k*	integer [2, 8]
Learning rate	*η*	log-uniform [10^−4^, 10^−2^]
Batch size	*B*	integer [32, 128]
Dropout rate	*p*_dropout_	real [0.1, 0.5]
Sequence length	*T*_seq_	integer [20, 100]

**Table 12:** Hyperparameter optimization space for the transformer baseline

Parameter name	Symbol	Optimization range
Feature dimension	*d*_model_	integer [16, 64]
Number of encoder layers	*N*_layers_	integer [1, 4]
Learning rate	*η*	log-uniform [10^−4^, 10^−2^]
Batch size	*B*	integer [32, 128]
Dropout rate	*p*_dropout_	real [0.1, 0.5]
Sequence length	*T*_seq_	integer [20, 100]

#### Optimal hyperparameters

The complete set of optimized hyperparameters for all models (BINN-ESN, standard ESN, LSTM, TCN, and Transformer), across all five systems (Lorenz, Chen, Lü, Mackey-Glass, and ETTh1) and all 10 independent trials, is available in the public repository. Specifically, the directory params_optimized/ contains one JSON file per model per system per seed (e.g., BINN_Lorenz_seed0.json), where each file records the full hyperparameter configuration identified by Bayesian optimization. The directory results_xxx/ further contains the corresponding evaluation metrics (MSE and VPT) for each trial.

Given the large volume of parameter configurations (5 systems × 5 models × 10 trials), we do not reproduce them exhaustively in this appendix. Instead, Tables 13 and 14 report the mean and standard deviation of each hyperparameter across the 10 trials for the BINN-ESN model, providing a compact overview of the optimized parameter distributions. The complete per-trial values can be accessed in the repository.

**Table 13:** Optimized hyperparameters for BINN-ESN across all systems (mean ± std over 10 seeds). Part I

System	***N***_***res***_	Input scale	Sparsity	Spectral radius	***α***_***f***_	***α***_***l***_	***α***_***e***_
Lorenz	1127 ± 283	1.2237 ± 0.4302	0.3196 ± 0.1316	0.6302 ± 0.3036	0.5647 ± 0.2631	0.6647 ± 0.2384	0.6973 ± 0.2669
Chen	1170 ± 325	1.2639 ± 0.6909	0.2526 ± 0.1472	0.6551 ± 0.4617	0.5147 ± 0.3167	0.5826 ± 0.2713	0.4261 ± 0.3046
Lü	1106 ± 260	1.1881 ± 0.5122	0.2139 ± 0.1305	0.6017 ± 0.4123	0.5484 ± 0.2581	0.6039 ± 0.2394	0.6541 ± 0.2003
Mackey-glass	1183 ± 280	1.2010 ± 0.5870	0.2585 ± 0.1414	0.7133 ± 0.3795	0.6549 ± 0.2787	0.5003 ± 0.2335	0.7668 ± 0.2219
ETTh1	1048 ± 297	1.2057 ± 0.5832	0.2616 ± 0.1697	0.8092 ± 0.2602	0.4053 ± 0.2630	0.3378 ± 0.2423	0.3630 ± 0.2382

**Table 14:** Optimized hyperparameters for BINN-ESN across all systems (mean ± std over 10 seeds). Part II

System	***β***	***V***_***F***_	***V***_***L***_	***V***_***E***_	***a***	reg
Lorenz	6.75 ± 2.85	0.57 ± 0.37	0.64 ± 0.35	0.40 ± 0.40	0.69 ± 0.24	6.32 × 10^−4^ ± 8.03 × 10^−4^
Chen	6.61 ± 2.70	0.46 ± 0.42	0.63 ± 0.34	0.47 ± 0.33	0.78 ± 0.26	3.55 × 10^−3^ ± 4.25 × 10^−3^
Lü	6.59 ± 2.26	0.34 ± 0.32	0.60 ± 0.38	0.37 ± 0.34	0.66 ± 0.21	2.80 × 10^−3^ ± 2.76 × 10^−3^
Mackey-glass	5.51 ± 2.73	0.53 ± 0.34	0.50 ± 0.31	0.49 ± 0.30	0.67 ± 0.24	2.54 × 10^−3^ ± 2.93 × 10^−3^
ETTh1	4.39 ± 1.95	0.55 ± 0.34	0.69 ± 0.15	0.48 ± 0.31	0.54 ± 0.15	1.45 × 10^−5^ ± 4.14 × 10^−5^

### Quantification and statistical analysis

This section provides the experimental protocol for evaluating the proposed BINN-ESN model. To ensure an unbiased evaluation and reproducibility, the entire workflow, from data generation to model evaluation, is fully automated.

#### Benchmark datasets and data preparation

Three well-established chaotic dynamical systems were considered as benchmark datasets to evaluate the ability of the tested models to learn and forecast complex dynamics. The set of equations that govern each system is as follows.

##### The Lorenz System

(Equation 18) $$\left\{\begin{array}{cc} \dot{x} & =\sigma \left(y-x\right),\\ \dot{y} & =x\left(\rho -z\right)-y,\\ \dot{z} & =xy-\beta z, \end{array}\right)$$

with the classic parameters set to *σ* = 10, *ρ* = 28, *β* = 8/3, and a step size of Δ*t* = 0.005.^41^

##### The Chen System

(Equation 19) $$\left\{\begin{array}{cc} \dot{x} & =a\left(y-x\right),\\ \dot{y} & =\left(c-a\right)x-xz+cy,\\ \dot{z} & =xy-bz, \end{array}\right)$$

with parameters of *a* = 35, *b* = 3, *c* = 28, and a step size of Δ*t* = 0.005.^42^

##### The Lü System

(Equation 20) $$\left\{\begin{array}{cc} \dot{x} & =a\left(y-x\right),\\ \dot{y} & =-xz+cy,\\ \dot{z} & =xy-bz, \end{array}\right)$$

with parameters of *a* = 36, *b* = 3, *c* = 20, and a step size of Δ*t* = 0.005.^43^

##### The Mackey-Glass System

To evaluate the model’s capability in handling delay differential equations (DDEs), we employ the Mackey-Glass system, a standard benchmark for chaotic time-series prediction.^44^ Its dynamics are governed by:

(Equation 21) $$\dot{x}\left(t\right)=\beta \frac{x\left(t-\tau \right)}{1+x{\left(t-\tau \right)}^{n}}-\gamma x\left(t\right)$$

We adopted the canonical chaotic parameters *β* = 0.2, *γ* = 0.1, *n* = 10, and a time delay *τ* = 17. The data was generated utilizing a fourth-order Runge–Kutta integrator with a step size of Δ*t* = 1.0. Consistent with the ordinary differential equation (ODE) systems, a sequence of 22,000 discrete points was synthesized.

##### Real-world dataset: ETTh1

To thoroughly demonstrate the generalizability and practical applicability of the BINN-ESN in noisy, non-stationary real-world environments,^13^ we further evaluated all models on the ETTh1 (Electricity Transformer Temperature) dataset https://www.kaggle.com/datasets/abiridir/etth1-dataset-csv. This widely recognized benchmark records the target oil temperature alongside six power load features. The dataset was strictly preprocessed to remove non-numeric identifiers, and missing values were imputed utilizing forward and backward filling strategies, framing it as a highly complex multivariate forecasting task. For this dataset, an 80-20 train-test split was consistently applied.

To ensure a rigorous, unbiased evaluation, we adopt a unified 10-trial experimental protocol for all models. For each trial, a distinct random seed (0 through 9) is used to generate the system trajectory, and the ODE systems are integrated via a fourth-order Runge–Kutta solver (solve_ivp) to produce 22,000 points. For synthetic chaotic systems (Lorenz, Chen, Lü, and Mackey-Glass), the first 2,000 points are discarded as washout to eliminate transient effects; the remaining 20,000 points are partitioned as follows: the first 18,000 points serve as the training set (which also contains the washout period that is discarded internally by ESN-family models), and the final 1,999 points are held out as the test set. Due to one-step input-target shift, effective test targets are 1,999 points. For the real-world ETTh1 dataset, a chronological 70%–30% train-test split is adopted, which is the standard practice in time-series forecasting literature^1^—unlike synthetic data, real-world data do not require a washout period because the recording already reflects the system’s steady-state dynamics. For all datasets, no data shuffling is applied at any stage to strictly preserve the temporal order, which is essential for both the teacher forcing phase and the free-running prediction phase. Hyperparameters are then optimized independently for each seed, yielding seed-specific parameter configurations and model checkpoints. This design is intentional: it tests whether performance remains reliable across different attractor regions induced by different initial conditions, rather than only under repeated fitting on a fixed trajectory. This protocol evaluates robustness under realistic compound uncertainty sources (trajectory initialization, hyperparameter search, and stochastic model initialization), rather than under fixed-data-only conditions. All the data are standardized to have zero means and unit variances using the StandardScaler of sklearn.

#### Baseline models for the comparative analysis

The performance of our proposed BINN-ESN is benchmarked against that of four established baseline models.

##### Standard echo state network (ESN)

The standard ESN characterized by the use of a static hyperbolic tangent activation function (tanh) with a leakage rate is considered here as the main reference. Its hyperparameter search space is detailed in Table 9. We intentionally selected the standard ESN as the primary baseline to strictly isolate the contribution of the proposed MCCNN neuron. By keeping the network topology (single reservoir layer) identical and only changing the neuronal dynamics, we demonstrate that the performance gain stems specifically from the bio-inspired internal dynamics, rather than from increased architectural depth or other topological modifications.

##### Long short-term memory (LSTM) network

The LSTM network was implemented via a standard multilayered LSTM architecture to constitute a baseline of the deep learning approach. Although newer architectures exist, LSTM remains a widely adopted standard baseline in time series forecasting literature. Benchmarking against LSTM provides a universally understood reference point for the trade-off between computational cost and predictive fidelity. Its key hyperparameters, including the network size and learning parameters, are also optimized via a Bayesian search, with the full search space provided in Table 10.

##### Temporal convolutional network (TCN)

To represent state-of-the-art convolutional architectures in time-series forecasting, a TCN model^45^ was included as a strong deep learning baseline. TCNs strictly utilize 1D dilated causal convolutions, which enable the network to achieve an exponentially large receptive field for capturing long-term dependencies while strictly preventing future information leakage. Furthermore, the architecture avoids the sequential bottleneck of standard RNNs, allowing for massive parallel computation. Its structural parameters, including the base number of channels and kernel sizes, were dynamically tuned via Bayesian optimization.

##### Transformer

Given the recent prominence of attention mechanisms across various domains, a Transformer^12^^,^^16^ was implemented to serve as a competitive sequence-to-sequence baseline. By integrating positional encoding and multi-head self-attention mechanisms, the Transformer dynamically models global temporal dependencies without relying on recursive unrolling. To ensure fair and robust optimization, an internal dimension interception mechanism was strictly engineered to prevent dimensionality conflicts during multi-head attention partitioning. Key structural hyperparameters, including the feature embedding dimensions, the number of encoder layers, and dropout rates, were comprehensively optimized.

#### Performance metrics

The performances of the tested models were evaluated through a set of quantitative metrics that are tailored for both short-term accuracy and long-term stability. All reported scores were calculated on the test set and presented as means ± standard deviations over 10 independent trials for all models, ensuring a fair and unified statistical comparison.

Let $Y={\left\{y\left(t\right)\right\}}_{t=1}^{T}$ be the sequence of true target vectors derived from the test set and $\hat{Y}={\left\{\hat{y}\left(t\right)\right\}}_{t=1}^{T}$ the corresponding sequence of predicted vectors generated by a model, where *T* is the total length of the test sequence.

##### Metrics for short-term accuracy

For single-step and short-horizon multistep prediction tasks, where the primary goal is precision, two standard error metrics were used.

##### Mean squared error (MSE)

The MSE measures the average squared Euclidean distance between the predicted and true vectors, providing greater sensitivity to larger deviations. It is defined as follows:

(Equation 22) $$\text{MSE}=\frac{1}{T}\overset{T}{\underset{t=1}{\sum }}{\left\|y\left(t\right)-\hat{y}\left(t\right)\right\|}_{2}^{2}$$

##### Mean absolute error (MAE)

The MAE measures the average L1 distance between the predicted and true vectors, providing a more direct measure of the magnitude of the average error. It is defined as follows:

(Equation 23) $$\text{MAE}=\frac{1}{T}\overset{T}{\underset{t=1}{\sum }}{\left\|y\left(t\right)-\hat{y}\left(t\right)\right\|}_{1}$$

##### Metrics for long-term stability

For long-term stability evaluation, a free-running (recursive) prediction task was employed. In this setup, the prediction produced by the tested model at time *t*, $\hat{y}\left(t\right)$, is used as the input for predicting the state at time *t* + 1, $\hat{y}\left(t+1\right)$. This process is repeated automatically for a fixed horizon of 2000 recursive prediction steps.

##### Normalized root mean squared error (NRMSE)

The NRMSE obtained by normalizing the standard root mean square error (RMSE) via the standard deviation of the true signal was used to provide a scale-independent error measure, thus allowing meaningful performance comparisons across different chaotic systems. It is calculated as follows:

(Equation 24) $$\text{NRMSE}=\frac{\sqrt{\frac{1}{T}\sum_{t=1}^{T}{\left\|y\left(t\right)-\hat{y}\left(t\right)\right\|}_{2}^{2}}}{\text{std}\left(Y\right)}$$

where std(**Y**) is the standard deviation computed over the entire true time series **Y**.

##### Valid prediction time (VPT)

The VPT is a metric that measures how long a model can autonomously generate a realistic trajectory before its predictions start to deviate significantly from the true path. It is defined as the number of time steps *t* during which a model can perform prediction in the free-running mode before the *cumulative* NRMSE (computed from step 1 up to step *t*) first exceeds a predefined threshold *τ*. In our investigations, a standard threshold of *τ* = 0.5 was used. A higher VPT indicates superior long-term stability.

##### ACF curve-based dynamical consistency

To evaluate whether model-generated trajectories preserve intrinsic temporal structure, we employ the Autocorrelation Function (ACF) curve. For a univariate time series *y*(*t*), the ACF at lag *k* is defined as:

(Equation 25) $$\text{ACF}\left(k\right)=\frac{\sum_{t=1}^{T-k}\left(y\left(t\right)-\bar{y}\right)\left(y\left(t+k\right)-\bar{y}\right)}{\sum_{t=1}^{T}{\left(y\left(t\right)-\bar{y}\right)}^{2}}$$

In our experiments, we compare ACF curves of model predictions against ground truth over lags 0–200. Closer overlap indicates better preservation of system timescales and dynamical consistency.

#### Computational environment

All the experiments were conducted on a high-performance computing server. The hardware of the server consisted of an Intel(R) Xeon(R) Gold 6248 CPU @ 2.50 GHz, 80 GB of DDR4 system RAM, and an NVIDIA Tesla V100 (32 GB) graphics processing unit (GPU) for accelerating the deep learning baseline experiments (LSTM, TCN, and Transformer).

The software environment was based on the Ubuntu 20.04.6 LTS operating system, and all the models and experiments were implemented in Python (version 3.9.23). The complete list of all the Python packages and their versions (as generated by conda) is provided in the publicly available code repository.

#### Commitment to reproducibility

All experiments were governed by a strict protocol to ensure reproducibility. Across 10 trials (seed 0–9), each trial independently performs trajectory generation, hyperparameter optimization, and model training/evaluation under its own seed-controlled stochastic pipeline. Therefore, trial-to-trial variability reflects the joint effect of data initialization, search stochasticity, and model initialization. All hyperparameters for all models were optimized automatically using the Bayesian optimization procedure detailed in Section STAR Methods. The complete code is publicly available at https://github.com/Jizhao-Liu/code-for-BINN-ESN.
